# Computational Assessment of a Dual-Action Ru(II)-Based Complex: Photosensitizer in Photodynamic Therapy and Intercalating Agent for Inducing DNA Damage

**DOI:** 10.1021/acs.inorgchem.3c00592

**Published:** 2023-05-29

**Authors:** Fortuna Ponte, Stefano Scoditti, Pierraffaele Barretta, Gloria Mazzone

**Affiliations:** Department of Chemistry and Chemical Technologies, University of Calabria, 87036 Rende, Italy

## Abstract

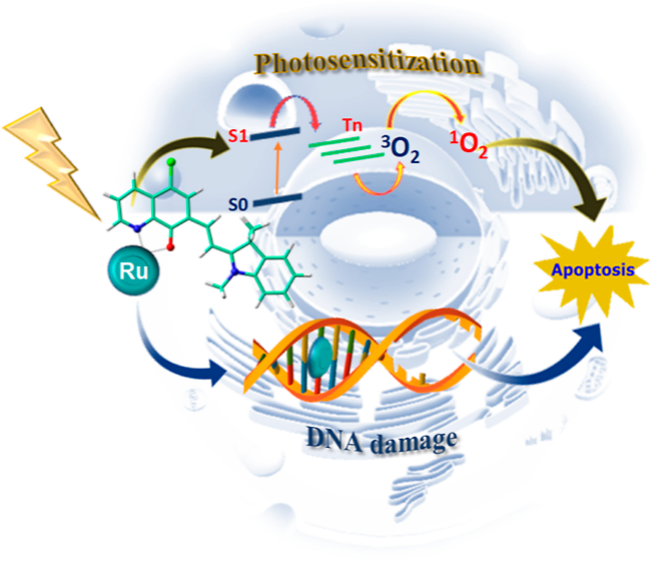

A combined quantum-mechanical and classical molecular dynamics study of a recent Ru(II) complex with potential dual anticancer action is reported here. The main basis for the multiple action relies on the merocyanine ligand, whose electronic structure allows the drug to be able to absorb within the therapeutic window and in turn efficiently generate ^1^O_2_ for photodynamic therapy application and to intercalate within two nucleobases couples establishing reversible electrostatic interactions with DNA. TDDFT outcomes, which include the absorption spectrum, triplet states energy, and spin–orbit matrix elements, evidence that the photosensitizing activity is ensured by an MLCT state at around 660 nm, involving the merocyanine-based ligand, and by an efficient ISC from such state to triplet states with different characters. On the other hand, the MD exploration of all the possible intercalation sites within the dodecamer B-DNA evidences the ability of the complex to establish several electrostatic interactions with the nucleobases, thus potentially inducing DNA damage, though the simulation of the absorption spectra for models extracted by each MD trajectory shows that the photosensitizing properties of the complex remain unaltered. The computational results support that the anti-tumor effect may be related to multiple mechanisms of action.

## Abstract 2

A combined quantum-mechanical and classical molecular dynamics study of a recent Ru(II) complex with potential dual anticancer action is reported here. Inspection of photophysical properties and structural features evidences the possibility of the complex to act as both singlet oxygen generator and intercalating agent.

## 1 Introduction

In the last years, ruthenium complexes have attracted much more attention as a new generation of metal-containing antitumor drugs.^1−4^ The major interest in such complexes started with the discovery of the antimetastatic properties of NAMI-A.^5,6^ Since then, several complexes exhibiting promising anticancer activity in cells, animals, and humans have been studied and proposed as effective metallodrugs.^7−9^ The potential application of Ru(II)-based drugs is not limited to the chemotherapy since some complexes exhibited application in another therapeutic practice, known as photodynamic therapy (PDT). This medical treatment requires the combined action of a chromophore, the photosensitizer (PS), the visible or near-visible light, and the molecular oxygen aimed at producing reactive oxygen species (ROS) which work as cytotoxic agents. The PDT mechanism involves the excitation of the PS from the ground state to a singlet excited one that, rather than decaying into the ground state through emissive phenomena, can transfer its energy, through a non-radiative intersystem spin crossing (ISC) process, to a triplet state. The lifetime of the triplet state longer than that of the singlet one allows energy or electron transfer to the surrounding molecules to generate the cytotoxic species. Production of such species can take place following two different photoprocesses: type I and type II. In the former pathway, the PS excited by light reacts, through an electron transfer process, with the surrounding oxygen molecules to yield ROS and inducing severe oxidative damage to cellular biological molecules (lipids, peptides, proteins, and nucleic acids) that are irreversibly degraded. In type II pathway, the triplet state directly transfers its energy to the molecular oxygen present in the environment to generate the highly cytotoxic ^1^O_2_ species.

To date, the only light-activatable Ru-based complex which entered into the human clinical trials is the octahedral complex TLD1433,^10^ proposed for the treatment of nonmuscle invasive bladder cancer. It is a Ru(II) polypyridyl complex containing a π-expanded ligand that ensures a prolonged triplet excited state lifetime of the type intraligand charge transfer (^3^ILCT) which slows competitive non-radiative transition back to the ground state. It is activated by green light irradiation and is able to generate ^1^O_2_ with high efficiency (quantum yield near to unity). In searching for more and more active Ru-based photosensitizers, also complexes potentially able to trigger DNA photo-cleavage have been synthesized and proposed as efficient anticancer agents. However, most of them are able to produce ROS upon irradiation with high-energy visible light,^11−14^ not falling within the therapeutic window (600–850 nm), the range of wavelengths within which tissue penetration is optimal. In recent years, some Ru(II) polypyridyl complexes have been designed for absorbing red or near-infrared light. Such a goal has been achieved modifying the structure of the ligands, such as substituting a bipy ligand with indolinepyridobenzopyran,^15,16^ using π-expanded tridentate ligands^9^ or appending a chromophore to a bipy ligand.^17^ However, other requirements for a photosensitizer to be considered photodynamically active have to be verified, such as the selectivity toward specific targets. The localization of the drug in different cellular compartments, such as mitochondria, lysosomes, nucleus, and so on, can, indeed, strongly influence the photodynamic therapeutic response, as competitive processes can reduce the PDT action.^18−20^

**Scheme 1:**
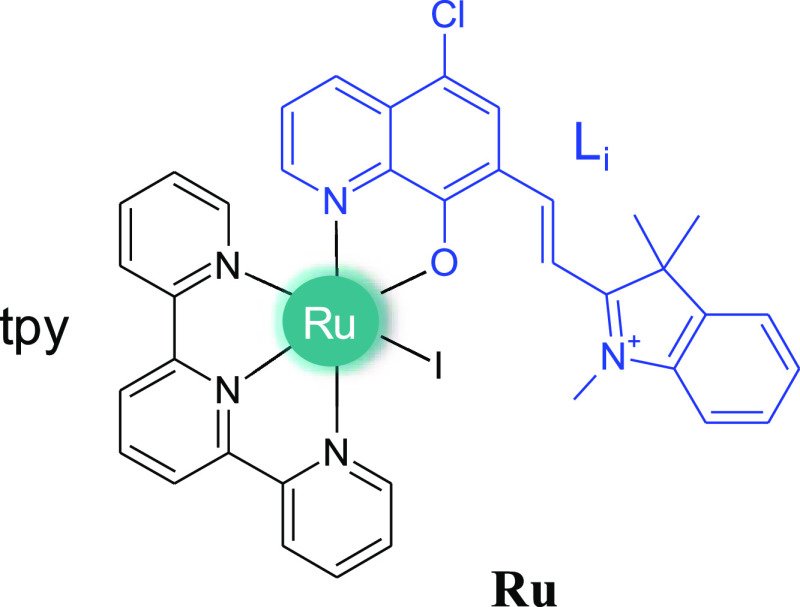
Schematic Structure of the Investigated **Ru** Complex [RuI(tpy)(L_i_)], with tpy = 2,2′;6′,2″-Terpyridine and L_i_ = 5-Chloro-7-[2-(1,3,3-trimethyl-3*H*-indol-1-ium-2-yl)vinyl]quinolin-8-olate, where the L_i_ Ligand is Represented in Blue

Depending on the chemical structure, beside the photogeneration of cytotoxic species, the action of the metal complexes as anticancer drugs can include reversible DNA interactions by intercalation. Such a process provides the establishment of non-covalent π–π stacking interactions between the drug and two base pairs in the DNA major and minor groove perpendicularly to the axis of the double helix without altering the stacking arrangement governed by Watson–Crick hydrogen bonding. Many anticancer drugs in clinical use possess a structure suitable for reversible interaction with DNA, as well as many chromophores, such as merocyanines, have been shown to possess intercalative properties and can thus be exploited in designing multi-functional cancer drugs.^15^ While many platinum complexes have been already proposed and computationally explored for DNA distortion by intercalation,^21−26^ only few octahedral ruthenium complexes have been suggested for this purpose,^16,27−30^ most probably because of their steric hindrance that do not facilitate the entrance of one of the coordinated ligands between base pairs to distort the DNA structure. In recent years, a merocyanine-based chromophore, the 5-chloro-7-(2-(1,3,3-trimethyl-3*H*-indol-1-ium-2-yl)vinyl)quinolin-8-olate, has been included in the scaffold of Ru(II) polypyridyl complexes for an efficient •OH-mediated DNA photocleavage upon red-light irradiation of low intensity.^15^ The electronic structure of the ligand, mainly responsible for red-shifting the maximum absorption wavelength of the polypyridyl ruthenium complex within the therapeutic window, should also allow the insertion of the drug between adjacent base pairs; moreover, the positive charge on one of its extremity should favor electrostatic interactions with the target. More recently, He et al. report the in vivo anticancer activity of a Ru(II)-based complex bearing the 2,2′;6′,2″-terpyridine (tpy) and the aforementioned chromophore, here labeled as the L_i_ ligand (Scheme 1), under red-light irradiation.^16^ It has been shown the selective accumulation of the Ru(II)-based drug into the lysosomes, one of the ideal target for PDT purposes. The authors have reasoned the choice to add L_i_ in the coordination sphere of the metal center essentially to have a red-shift absorption, no details about the possibility of such ligand to serve as intercalating agent^15^ have been provided.^16^ Nevertheless, such a complex could be, in principle, able to mediate the cytotoxic activity via either producing in situ the ^1^O_2_ cytotoxic agent by interaction with light or reversible interactions with DNA base pairs by intercalation, or both at the same time.

To ascertain the potential dual activity of the Ru complex reported in Scheme 1 and to explore the mechanism of action of such a complex, quantum mechanics and molecular dynamics (MD) simulations have been combined. Density functional theory (DFT) and its time-dependent extension (TDDFT) has been exploited to analyze the spectroscopic properties of the complex checking its ability to act as a photosensitizer in PDT. Classical MD approach has been, instead, used to study the mode of interaction with DNA. The resulted arrangements have been, then, investigated at quantum–mechanics level to detailing analyze the type of interactions established between the complex and DNA, comparing the behavior to that of the ligand alone. The effect on spectroscopic properties of the Ru complex upon intercalation has been also explored in order to check whether the non-covalent π–π stacking interactions of the complex with the base pairs could alter the photosensitizing properties of the complex after reversible DNA interaction.

## 2 Computational Details

The Gaussian 16 suite of program^31^ has been employed to perform all the quantum mechanical calculations in the framework of DFT and time-dependent DFT (TDDFT).

Ground singlet state optimization have been carried out by using the B3LYP functional^32,33^ with Grimme D3 dispersion correction^34^ and adopting the SMD implicit solvation model^35^ to simulate water as solvent (ε = 78). Stuttgart/Dresden effective core potential and corresponding split valence basis set^36^ have been used to describe Ru and I atoms, while 6-31G(d) basis set has been used to describe the rest of atoms.

To describe the photophysical properties and simulate the optical absorption spectra of the systems under investigation, TDDFT calculations, on the optimized ground state structures, have been performed. A preliminary benchmark study has been carried out and the comparison of the calculated maximum absorption wavelength with the corresponding experimentally detected for the **Ru** complex^37^ in water solvent has been performed. The computational results are summarized in Figure S1. Specifically, B3LYP-D3, B3PW91,^32,38^ CAM-B3LYP-D3,^39^ B97D,^40^ ϖB97XD,^41^ TPSS, PBE0,^42^ PBE,^43^ M05,^44^ M06,^45^ M06L,^46^ M11,^47^ MN12L,^48^ MN15,^49^ and MN15L^50^ functionals have been tested and M06 has been, accordingly, selected. The hybrid functionals are, indeed, well known functionals to study the photophysical properties of Ru polypyridyl complexes,^51−56^ and, in particular, the use of M06 in exploring the optical properties of a Ru(II)-based complex it has been shown to provide the best results if compared with outcomes coming from the second-order perturbation theory restricted active space (RASPT2/RASSCF) calculations.^57^ The whole absorption spectrum of **Ru** has been, thus, obtained as vertical electronic excitations from the optimized ground-state structure within the TD-M06 response theory by performing 150 electronic excitations. The same protocol has been used for computing the absorption spectra of ligands surrounding the metal center by performing 25 electronic excitations. TheoDORE software has been subsequently used to compute a fragment-based analysis for assigning state character.^58^

For the **Ru** system, to establish the occurring of ISC processes, the spin–orbit matrix elements for the coupling of the states potentially involved have been calculated at the ground state optimized geometry of the investigated systems with ORCA code^59^ and SOC values obtained according to eq 1
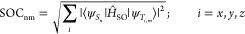
1where  is the spin–orbit Hamiltonian with effective nuclear charge. Relativistic corrections have been obtained by the zeroth-order regular approximation (ZORA). Thus, ZORA-DEF2-SVP and SARC-ZORA-SVP for the main and metal atoms, respectively, have been used. The RIJCOSX approximations has been introduced to speed up the calculations’ time, as suggested in the ORCA manual.

MD calculations have been carried out in order to study the intercalation of the ruthenium complex into DNA using a B-DNA dodecamer with Protein Data Bank code 1BNA.^60^ For the DNA system, hydrogens have been added to the model system using the H++ web-server.^61,62^ The **Ru** complex has been parametrized by using both Gaussian 16 and MCPB.py^63^ available in the Amber 16 package,^64^ following a procedure successfully applied to a similar system.^21−23^ Geometry optimization and frequency calculations have been carried out using Gaussian 16 using SDD effective core potential and split valence basis set to describe Ru and I atoms and 6-311G** basis set for the rest of the atoms. For the charges, the same software has been used to calculate the Merz–Kollman ESP charge of the created metal center model. Finally, MCPB.py has been, then, used to perform RESP charge fitting. The obtained parameters are reported in the Supporting Information. The drug has been manually introduced into the different potential intercalation sites. Topology and coordinate files have been generated by means of tleap in Amber 16 using the standard DNA.bsc1^65^ and GAFF force fields^66^ together with the newly generated parameters (reported in the Supporting Information) for the metal center obtained by the MCPB.py script present in Amber16. An octahedral box of water molecules around the system with a 14 Å buffer distance has been built using a TIP3P solvation model.^67^ In order to neutralize the system, Na^+^ ions have been added. 1000 minimization steps with a cutoff distance of 15.0 Å and a constant volume periodic boundaries have been carried out to relax the system before the MD. During this minimization, the DNA and the **Ru** complex have been fixed by using a force constant of 500 kcal mol^–1^. A second minimization (2500 steps) has been carried out without restrain with the same cutoff distance and constant volume periodic boundaries. Subsequently, the system has been heated to 300 K over 10000 steps for a total of 20 ps. SHAKE algorithm has been implemented to constrain bonds involving hydrogen. During the heating, the DNA and **Ru** complex have been weakly restrained by a force constant of 10 kcal mol^–1^ while keeping constant volume periodic boundaries and same cutoff distance. Equilibration followed by production of MD for 100 ns at 300 K have been ran under similar conditions with a 2 fs interval with no restraints. Binding free energy between **Ru** complex and DNA has been evaluated by the MM-PBSA method,^68^ as implemented in MMPBSA.py script in Amber 16 package, considering the whole production trajectory over 100 ns. Cpptraj in the Amber 16 package has been used to analyze the trajectories^69^ and VMD program to generate the figures out of the dynamics trajectory.^70^

Cluster analysis has been performed to obtain the most representative structure for each MD simulation. A cut of the obtained arrangements has been, thus, effected to analyze the specific interactions between the drug and the nucleobases surrounding it. Non-covalent interactions (NCIs) have been investigated by using the method proposed by Johnson et al.,^71^ RDG (reduced density gradient) analysis, using the Multiwfn 3.8 software.^72^ For each intercalation site, a model constituted by the drug and the eight surrounding nucleotides has been built. They have been used for further quantum-mechanical calculations, in which the drug and the four nucleobases around have been optimized, while the peripheral nucleotides have been kept frozen.

Subsequently, TDDFT calculations have been performed to simulate the absorption spectrum upon intercalation using the same computational protocol employed for obtaining the absorption spectrum of the complex alone.

## 3 Results and Discussion

The investigation of the mechanism of action of the **Ru** complex has been planned to clarify the structural and electronic features affecting its action as a cytotoxic agent. This includes the possibility to act as photosensitizer as well as to induce DNA damage by intercalation. For the two activities, the proper computational protocol has been selected; the photophysical properties accounting for photosensitizing action has been explored by means of DFT and its time-dependent formalism, while the propensity of the **Ru** complex to reversibly interact with DNA has been clarified by classical MD simulations, which required the parametrization of the complex. As stated in the Computational Details, the procedure followed for parameters generation (see the Supporting Information) has been successfully applied to a similar system.^21−23^

### 3.1 Photosensitizing Properties of **Ru** Complex

To exert the photosensitizing action in the therapeutic practice, a photosensitizer must possess a series of chemical and photophysical properties that ensure the selectivity and the effectiveness typical of PDT. These include an easy and reproducible synthesis, a good chemical stability and solubility in aqueous media as well as a good accumulation into the target tissues. Moreover, it has to be not toxic in the dark, to absorb within the therapeutic window (600–850 nm) and to have high quantum yield of the triplet excited state. As pointed out in the Introduction, the PDT requires, once the drug is administrated, the irradiation of the tissue with light of proper wavelength (therapeutic window). In this way, the photosensitizer transits from the ground to an excited singlet state, from which a radiationless transition from the S_n_ to T_m_ state could occur if the relativistic effect spin–orbit coupling is significant. These photophysical properties can be computationally evaluated by simulating the absorption spectra, locating the excited triplet state and determining the amplitude of the spin–orbit coupling matrix elements accounting for the radiationless ISC processes accessible to the **Ru** complex, as reported for several chromophores.^21,51,53,73−76^

**Figure 1:**
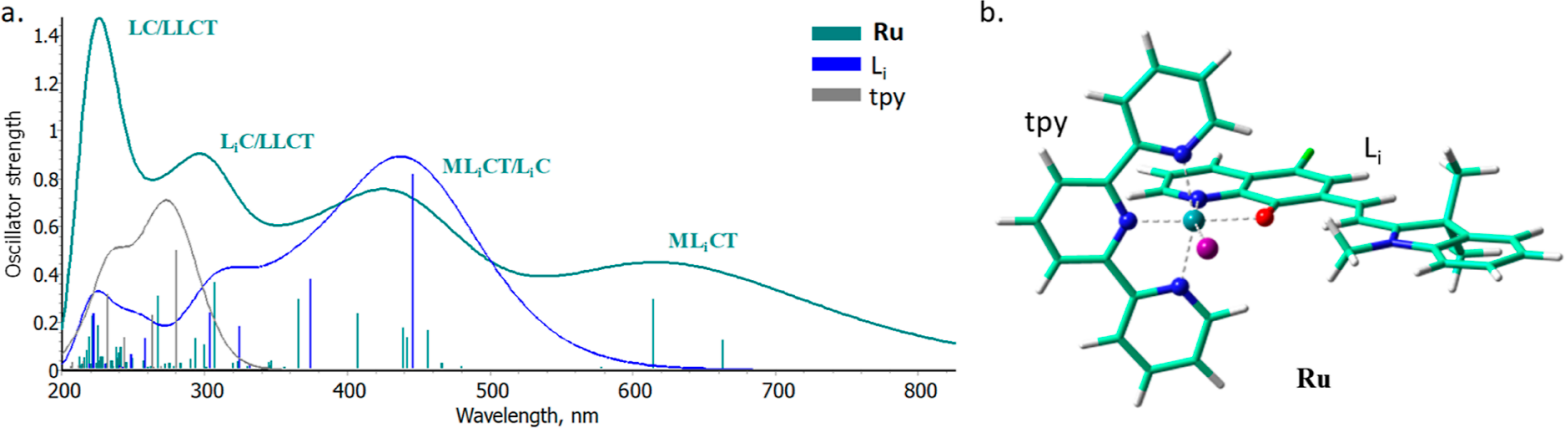
(a) M06 computed absorption spectra of the free tpy (gray line) and L_i_ (blue line) ligands and **Ru** complex (teal line). The Gaussian convolution of the spectra is accomplished with a half-width of 0.33 eV. (b) B3LYP-D3 optimized structure of **Ru** complex.

In order to describe as properly as possible, the photophysical properties of the complex, a preliminary benchmark study has been carried out on the maximum absorption wavelength and reported in Figure S1. Several exchange and correlation functionals have been taken into consideration and from such exploration, the global hybrid functional M06 emerged as the best choice for reproducing the electronic spectrum of **Ru** complex. Thus, the computed spectra of the complex and ligands, L_i_ and tpy, are reported in Figure 1, where the theoretical assignment to the most significant bands observed in the complex’s spectrum is included. The B3LYP-D3 optimized structure of the **Ru** complex is reported in Figure 1 as well. Detailed information about each electronic transition have been, instead, collected in Table S1. At a first glance, the comparison between them evidences the appearance of a band in the **Ru** complex not imputable to the ligands. Indeed, while the maximum absorption of L_i_ is found at around 450 nm, the complex exhibits the maximum absorption around 660 nm. The resulted band is originated by two electronic transitions, namely, states S1 and S4 in Table S1, HOMO (H) → LUMO (L) and H–1 → L at 662 and 614 nm, respectively. From the decomposition analysis (Figure S2) performed and the inspection of the NTO plots (Figure S3), the major contribution on such transitions entails a charge transfer from an orbital primary localized on the metal, and in part to tpy and iodine ligands, to an orbital mainly outspreaded on the L_i_ ligand, generating a metal-to-ligand charge transfer ML_i_CT band. The higher energy bands, instead, follow the profile of the two ligands. First that of L_i_ (in the region between 300 and 450 nm), the associated electronic transitions, indeed, evidences a substantial ML_i_CT, though a certain contribution of CT localized on L_i_ can be observed as well, making the whole band with mixed character ML_i_CT/L_i_C (Figure S4). Such a band is similarly well reproduced by computations, as it is centered at 440 nm, similarly to that recorded.^16^ The highest energy absorption bands, III and IV, instead, are generated by electronic transitions involving both tpy and L_i_ ligands (200–350 nm). Indeed, the convolution of the absorption spectrum in this region shows an increase in the intensity because of the contribution of each ligand to the transition of the other. Accordingly, while the band III is primary localized on the L_i_ (L_i_C), in the band IV charge transfer localized on the tpy ligand can be observed (LC) (see Figure S4). In both cases, a substantial contribution of a LLCT can be observed, generating again bands with a mixed character.

**Figure 2:**
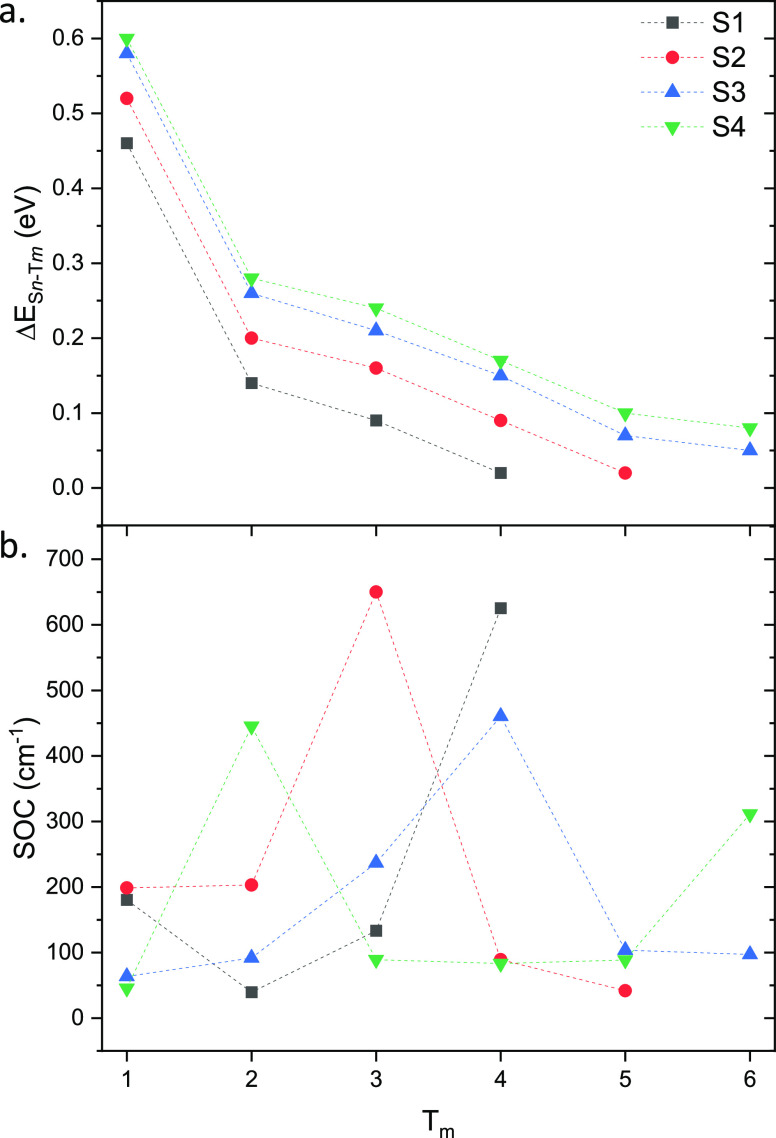
Computed (a) singlet–triplet splitting energies, Δ*E* (eV), and (b) SOCs (cm^–1^) for S*n*–T*m* (with *n* = 1–4 and *m* = 1–6) radiationless transitions between the involved states. All of the values are collected in Table S3.

The excitation to the bright singlet state should be followed by the radiationless transition to a triplet state lying close in energy to the bright one. From here, the photosensitizer can generate ROS by either transfer its energy or an electron, in a direct or indirect manner, to molecular oxygen. It has been reported that **Ru** is able to generate only ^1^O_2_ as cytotoxic agent, no other ROS have been experimentally detected.^16^ This means that only type II reactions are accessible to the **Ru** complex. Therefore, the low-lying triplet state has to have enough energy to be able to excite molecular oxygen transferring its energy to ^3^O_2_ and generating the cytotoxic agent ^1^O_2_. The energy splitting between the ground triplet (^3^Σ_g_^–^) and the excited singlet states (^1^Δ_*g*_) of molecular oxygen has been previously computed to be between 0.90 and 0.93 eV,^51,77−79^ in good agreement with the experimental value of 0.98 eV. The used computational protocol that better fits that used in this work returns a value of 0.90 eV,^78^ accordingly, it has been taken as reference to establish that the low-lying triplet state of **Ru**, located at 1.42 eV (Table S2), is able to excite molecular oxygen by transferring its energy. As two electronic transitions mainly contribute to the first absorption band, namely, S1 and S4, all of the plausible couplings between S1–S4 states and the triplet states lying below, T1–T6, have been calculated and reported in Figure 2, where also the energy differences between the coupled states have been provided. It is well-known, indeed, that the main parameters from which the ISC kinetics depends on are as follows: (i) the spin–orbit coupling elements and (ii) the singlet–triplet splitting energies.

As it can be inferred from these data (Figure 2b), whatever the triplet state potentially involved in the ISC, all of the singlet states deactivation channels can be highly probable. The computed SOC values are, indeed, much higher than typical chromophore entered in different phases of clinical trial, though in line with other Ru-based complexes.^51−53,76^ This is due to the well-known heavy atom effect accomplished by the metal center. However, paying attention also to the energy splitting between the coupled states is unreasonable that, e.g., T1 state can be populated by direct ISC from whatever the starting singlet state as it lies more than 0.4 eV lower in energy than the coupled state. While it is more probable that T3 and T4 could be involved in the ISC, as their couplings are characterized by high SOC values and small energy gap with the coupled states, i.e., S3 or S4. Therefore, the low-lying triplet state could be populated through internal conversion from the higher triplet states. Moreover, looking at the decomposition analysis (Figure S5) as well as at the NTOs (Figure S6) of triplet states it can be noted that the highest SOC values have been obtained for the coupling between states with different characters, e.g., S2 and T3, though both are mainly characterized by a CT from the M to the ligand, the involved orbitals are outspreaded on different portions of the molecule, i.e., L_i_ and tpy ligands in the two cases, respectively.

Alongside type II pathway, other photoprocesses, referred as type I, involving both ground, indicated as [Ru]^+^, and triplet state, ^3^[Ru]^+^, of the PS could in principle occur once the triplet state is populated. Actually, He et al. have been observed that no other types of ROS other than singlet oxygen are produced by **Ru** complex.^16^ Our computations, that are vertical electron affinity (VEA) and ionization potential (VIP) collected in Table S4, confirm that these reactions cannot be triggered by light in **Ru** complex. Indeed, as previously reported^53^ for autoionization reactions to occur, the proclivity of the PS to lose an electron has to be in favor of the excited triplet state instead of the photosensitizer in the ground state, which has to acquire the electron. Comparing VEA and VIP of [Ru]^+^ and ^3^[Ru]^+^, it can be seen that the only accessible pathway is that involving the electron transfer between two neighboring ^3^[Ru]^+^, process b (Table S4). However, such reaction should be followed by a favorable electron transfer from the formed radical to the triplet molecular oxygen to yield the active ROS O_2_^•(−)^ (process d). Being VEA of [Ru]^•^ [that is equal to −VEA([Ru]^+^)] greater than VEA (^3^O_2_) the process cannot be favored. Even the direct production of O_2_^•(−)^ (process c in Table S4) by electron transfer from the ^3^[Ru]^+^ to molecular oxygen is inaccessible because of the greater absolute value of VIP (^3^[Ru]^+^) with respect to VEA (^3^O_2_). Hence, **Ru** can act only as ^1^O_2_ generators according to type II reactions.

### 3.2 **Ru** Intercalation Binding to DNA

As stated in the Introduction, though not explicitly taken into consideration in the experimental exploration,^16^ the structural features of the merocyanine scaffold allows the complex to be inserted between two DNA base pairs. In this section, the intercalation process of the **Ru** complex with the duplex DNA will be discussed. Clustering analysis has been used to extract the most representative conformations of the complex intercalated into DNA. In particular, RMSD of four surrounding base pairs has been used as parameter for clustering. The most representative structures extracted out of the clustering analysis have been cut to obtain a model for each intercalation site to be used for further QM calculations, including the drug and the four surrounding nucleotides. Thus, the photophysical behavior of **Ru** complex upon intercalation and a detailed analyses of the interactions put into play in such adducts’ formation have been investigated. Due to the nature of the formed intercalation adducts, in which NCIs play an important role, RDG analysis have been carried out to determine the characteristics of non-covalent intermolecular interactions.

#### 3.2.1 Intercalation Adducts and Binding Energies

In the last few years, polypyridyl ruthenium(II) complexes have been extensively investigated as potential anticancer agents.^80^ The anticancer activity of the ruthenium-based compounds involves the interaction with DNA, through processes of intercalation, electrostatic interaction, by binding through minor or major grooves, as well as combinations of all these modes.^81^ The intercalation action is one of the most important mechanisms carried out by these complexes. The insertion occurs without disturbing the overall stacking pattern due to the Watson–Crick hydrogen bonding between the two strands. Such a process starts with the transition of the intercalating agent from the aqueous environment to the hydrophobic space between two adjacent DNA base pairs. The environment can be, thus, simulated by explicitly considering water as solvent.

The **Ru** complex here investigated is structurally suitable to be able to interact with DNA by reversible intercalation mode, thanks to the planarity and aromaticity of the large-conjugated ligand L_i_, in which the 5-chloro-8-oxyquinolate, the quinoline moiety here named **quin** is incorporated into a merocyanine scaffold linked to a vinyl indole-based fragment, here labeled as **ind**.

**Scheme 2:**
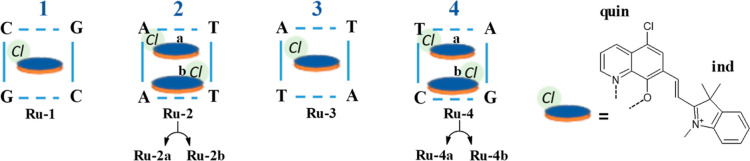
Schematic Representation of **Ru** Complex Intercalated at the Four Possible Positions, Named **1–4** of the B-DNA Dodecamer

The MD simulations have been planned in order to consider all the possible intercalation sites in the major groove of the B-DNA dodecamer for the **Ru** complex in analogy with our previous works.^21−23,82^ The drug has been, thus, manually placed in the intercalation sites, here labeled as 1, 2, 3, and 4, by introducing the L_i_ portion of the complex. The examined systems in which **Ru** is included between DNA base pairs are shown in Scheme 2, where the arrangements indicated as **Ru-1**, **Ru-2**, **Ru-3**, and **Ru-4** correspond to the structures CG/**Ru**/GC, AT/**Ru**/AT, AT/**Ru**/TA, and TA/**Ru**/CG, respectively. Because of the asymmetric nature of the L_i_ ligand, two different orientations of the drug within the sites **2** and **4** have been taken into consideration. The resulted arrangements have been called **a** or **b** when the chlorine of the **quin** part points to the left or to the right filament, respectively.

A 100 ns long MD simulation has been performed for the six models just described. Time evolution of root mean squared deviation (RMSD) calculated from the dynamics run, for all the examined situations, can give an indication of conformational changes induced by intercalation. An increasing of the RMSD values for a particular system generally indicates a conformational change during the MD simulation. The plots of RMSD, considering the DNA, the four residues around **Ru** complex and the intercalated drug for each MD run, are reported in Figure S7. In all the investigated systems, the RMSD value for the whole DNA dodecamer is very similar throughout the dynamic trajectory, while there is a clear variation in the RMSD relative to the four residues considered for the **Ru-1** and **Ru-2b** systems. Such an increase in these two systems indicates that the active portion of DNA is subjected to considerable structural changes induced by the presence of the drug. In particular, the intercalation adduct formation increases the spacing between the base pairs belonging to the intercalation site.

To estimate the stability of the Ru-DNA adducts, the MM-PBSA approach has been applied to all the MD simulations. The resulted binding free energies between the DNA and **Ru** complex have been collected in Table S5 and are all of the same order of magnitude. As expected, a favorable intercalation of **Ru** complex within the DNA major groove has been observed, being negative all the calculated values. The free energy values are comprised between −4.0 and −9.9 kcal mol^–1^, obtained for **Ru-4a** and **Ru-1**, respectively. A similar value to that obtained for **Ru-1** has been found in the case of the **Ru-2b** system. Therefore, **Ru-1** adduct, in which the **Ru** complex is intercalated in the site **1** (CG/GC), is found to be the most stable, followed by the **Ru-2b** system, that involves AT/AT base pairs.

**Figure 3:**
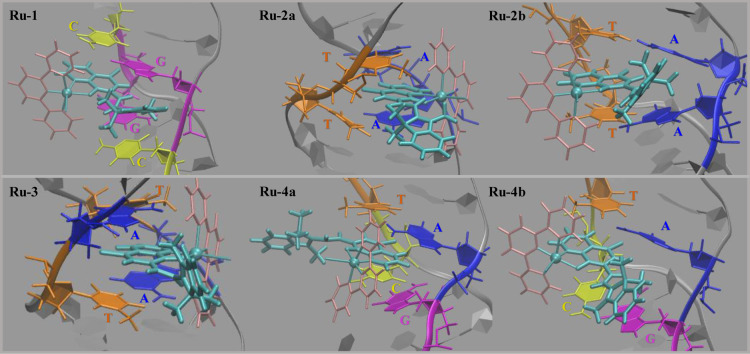
Most representative structures of **Ru** complex intercalated at positions **1–4** out of clustering analysis performed on the 100 ns long MD.

All the trajectories have been subjected to the cluster analysis in order to obtain the most representative structure of the whole simulation for each intercalation site, which are reported in Figure 3, to use for further quantum mechanical calculations. At a first glance, it can be noted that in the two systems aforementioned, the drug is totally included (**Ru-1**) or partially included but very close (**Ru-2b**) to the inclusion site, differently from the other situations, in which the intercalating ligand L_i_ occupies a marginal place with respect to the base pairs site hosting it, especially with the charged **ind** portion. This behavior can be an explanation of the RMSD values observed for the residues of these systems and not for the others.

In all the intercalations sites, the interaction of the drug with DNA essentially occurs through the **quin** part of the L_i_ ligand and not the whole merocyanine ligand. Only in **Ru-1** arrangement, the **ind** fragment is also included into the active intercalation site, playing essential role in the stabilization of the structure. From Figure 3, it is also possible to observe that in all the obtained intercalated states, while the **quin** ring is inside the intercalation site, the metal atom together with tpy and iodide ligands are totally outside the DNA active sites. In **Ru-1** adduct, where L_i_ ligand is completely lodged into the intercalation site (CG/GC), **quin** and **ind** fragments are coplanar. Looking at **Ru-2a** and **Ru-2b**, instead, in the most representative structures included in Figure 3, the drug is placed into the AT/AT site with the **quin** ring, while the **ind** portion in the former lies completely outside of the DNA base pairs, in the latter it is very close to the active site establishing interactions with the adenine nucleobase. In both the arrangements, the two fragments of the L_i_ ligand are arranged almost parallel among themselves. In **Ru-3** and **Ru-4a**, instead, it is evident that both **quin** and **ind** fragments are no longer coplanar one to the other (the measured torsion angle is about 50°) with the **quin** portion included in the base pairs cavity and the **ind** group is entirely outside. **Ru-4b** arrangement shows the inclusion of the L_i_ ligand essentially with the **quin** part; **ind**, though not included in the intercalation site, remains very close to the active site.

Overall, the MD outcomes show a non-conventional intercalation of **Ru** within the B-DNA as the intercalative agent occupy the site essentially with the **quin** fragment and only in same cases with the inclusion of the **ind** portion as well. Comparing the obtained arrangements with those reported in literature, it can be also noted that the steric hindrance of the complex, especially the tpy ligand, prevents a greater penetration of the complex within the intercalation site. Indeed, in all cases an interaction of the tpy with the closest bases can be observed. In addition, such hindrance entails an opening of the intercalation site from one side, generating something like a pocket, instead of an enlargement of the entire site often observed for other intercalative agents.^21−23,82,83^

However, as already drawn from the RMSD plot of the four residues defining each intercalation site, analyzing the whole trajectory, the opening of the various intercalation sites occurs during the intercalation process. Specifically, the intercalation causes an elongation of about 3 Å of the DNA double helix, in each investigated drug-DNA adduct. The obtained energy values for the **Ru-1** system is consistent with a deeper drug inclusion, as shown by the intercalation geometry reported in Figure 3. In **Ru-2b**, the proximity of **ind** part to the DNA bases contributes in stabilizing the system similarly to **Ru-1** (−9.6 vs −9.9 kcal mol^–1^, respectively), where the ligand is totally lodged in the intercalation site. As in all the individual intercalation adducts, the **quin** group is lodged between the four nucleotides, the energy differences in the various arrangements can be arguably attributed to the distance of the **ind** fragment from the intercalation site of the DNA and to its orientation. Interestingly, a greater perturbation to the energy and consequently a destabilization of the system is observed when the **ind** portion is far from the coplanarity with the portion **quin**. This means that when the **quin** and **ind** fragments are coplanar, a better electronic communication in the ligand is ensured thus obtaining a greater interaction with the DNA base pairs and, then, a major stability of the drug-DNA adduct.

Although the inclusion process of **Ru** takes place, the calculated binding energies are not as negative as indicated for other intercalating agents reported in the literature.^21,22,82^ Therefore, the investigation has been extended to the L_i_ ligand alone, with the aim of understanding whether the steric hindrance generated by the coordination to the metal center could influence the intercalation properties of the merocyanine and therefore the adducts’ stability. Among all the investigated intercalation sites, the inclusion of the L_i_ has been tested in the site with which **Ru** complex shown the grater interaction, that is, CG/GC intercalation site (**1**). Detailed information on the results of the 100 ns long MD simulations are available in Figures S8 and S9 of the Supporting Information. The simulation confirms that the L_i_ structure is compatible for stacking between DNA bases. Again, the key portion accounting for DNA interaction is the **quin** fragment, while the positively charged fragment of the merocyanine scaffold, **ind**, establishes only a few interactions with the bases surrounding, similarly to most of the explored situations with the **Ru** complex interacting with DNA. The calculated binding energy equal to −9.4 kcal mol^–1^ evidences the same propensity of the ligand alone to reversible interact with DNA by intercalation, as the value is similar to that calculated when it is coordinated to the ruthenium center in the CG/GC site.

#### 3.2.2 QM Models: Structural Features and Absorption Spectra

**Figure 4:**
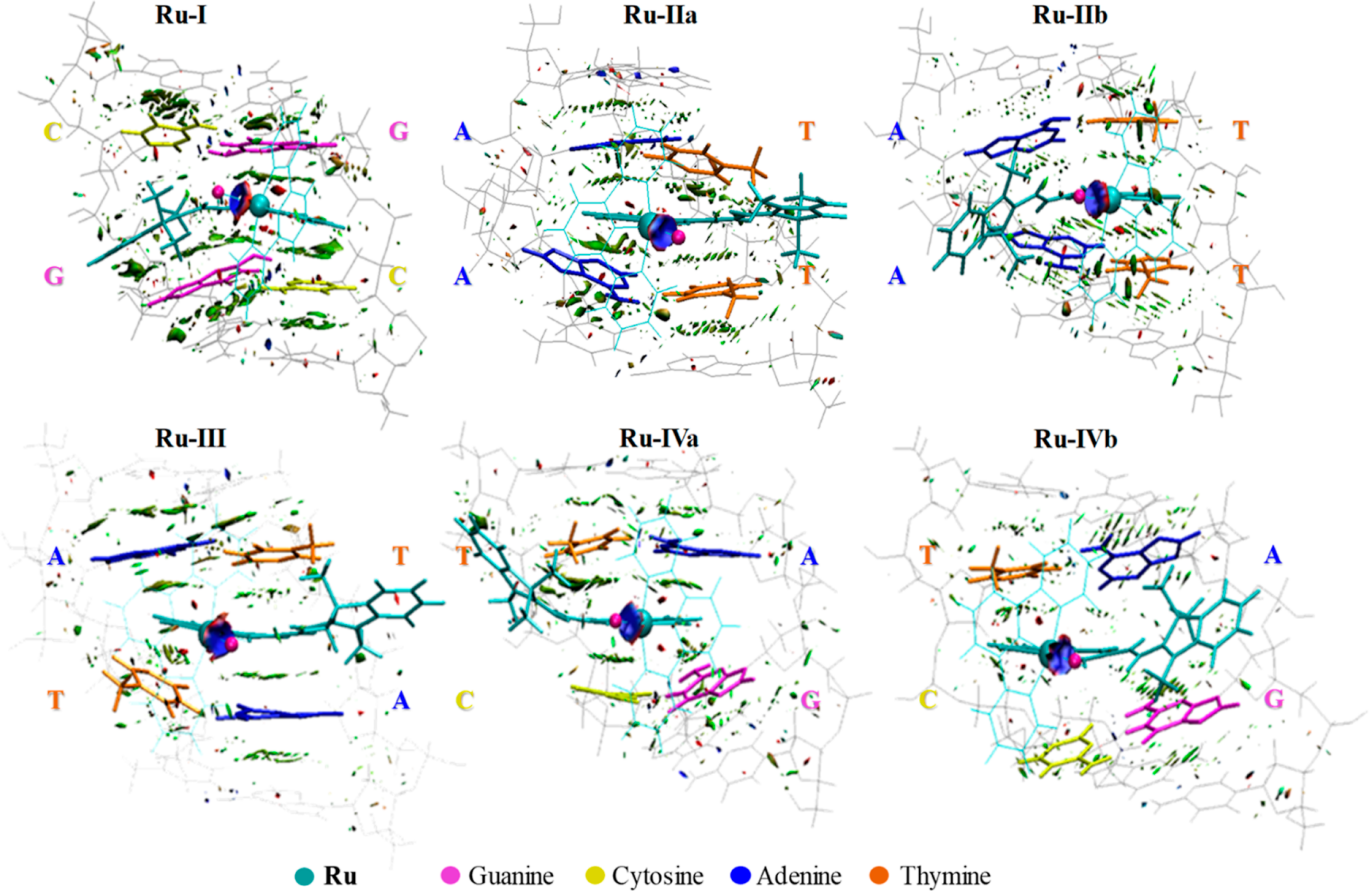
RDG iso-surface map (with RDG = 0.5 isovalue) calculated for the optimized geometries of the representative structures extracted from the most populated cluster selected from each MD simulation that include **Ru** complex and the eight surrounding nucleotides for the six investigated intercalated positions. The bases pairs not participating in the π–π interactions and the tpy ligand of the complex are represented in lines, Ru complex and the surrounding four nucleotides, identifying the intercalation sites, are displayed in tubes and balls.

In order to analyze more in detail, the specific interactions accounting for the different stability of the considered adducts and to establish the influence of DNA on the photophysical properties of the intercalated PS, smallest models have been built. They have been derived from the most representative structures of each MD simulation and include the drug and the eight surrounding nucleotides. Because of the absence of the rest of the dodecamer used for the MD intercalation study, to avoid the unrealistic distortion of the DNA portion considered in the QM calculations, the optimization has been limited to the intercalated complex and the two bases pairs defining the specific intercalation site, while the Cartesian coordinates of the other DNA pair bases have been kept frozen. To distinguish such models from the MD structures described above, the six models have been labeled replacing **1**–**4** with **I**-**IV** indices, thus obtaining structures **Ru-I**, **Ru-IIa**, **Ru-IIb**, **Ru-III**, **Ru-IVa**, and **Ru-IVb**. Thereafter, an additional analysis of NCI between the intercalator and DNA portion has been performed by using the method proposed by Johnson et al.,^71^ NCI-RDG analysis. In Figure 4, the optimized structures of the most stable geometrical arrangements and the graphical visualization of NCIs obtained by NCI-RDG analysis are reported. From the analysis of the colored RDG map, the intramolecular regions are dominated by the color green, associated with delocalized weak interactions and π-stacking interaction are expected. The intensity of the green color is associated to a stronger interaction.

As expected, in all the optimized structures, the intercalated ligand L_i_ plays the major role in the interaction with DNA base pairs. Though the ligand is essentially always characterized by a coplanarity of the two portions, its orientation in the active sites does not change so much compared to that observed in the unrelaxed most representative structures out of MD simulations. In all the investigated systems, π–π stacking interactions between L_i_ are clearly evidenced by the isosurfaces filling the interlayer spaces. In particular, in the **Ru-I** system, the **quin** ring strongly interacts with cytosine and guanine residues anchored to the single-stranded region of DNA, while the **ind** fragment interacts more specifically with the guanine nucleobase of the other filament. Moreover, π–π interactions between L_i_ double bond and the guanine can be also observed.

Looking at the **Ru-IIa** adduct, only the **quin** aromatic ring, lodged in the center of the intercalation site (AT/AT), interacts strongly with the two adenine bases and mildly with the thymine bases on the opposite strand. The **Ru-IIb** arrangement shows important π–π interactions between the **quin** group and both the adenine bases and also the double bond interaction with the two thymine nucleobases is clearly visible, with positively charged group, **ind**, very close to the active site. In the **Ru-III** system, **ind** fragment along with the C=C double bond are far from the active site, while the **quin** portion is centered within the intercalation site. Accordingly, few interactions with the four nucleobases may be highlighted. Also in **Ru-IVa** adduct the **quin** ring is located between the TA/CG base pairs and mirrors the same situation as the **Ru-III** arrangement. Finally, the **Ru-IVb** system exhibits a weak interaction between **quin** and thymine, while more intense interactions between the double bond and guanine and CH_3_–π interactions adenine and guanine can be observed.

These outcomes revealed that π–π stacking interactions between the intercalative agent L_i_ and the base pairs of the active intercalation site governs the intercalation process. Moreover, the analysis of the interactions confirms that the tpy ligand prevents a deeper penetration of the intercalative agent within the specific site, as in all cases, it establishes an interaction with the nucleotides in its proximity. Specifically, the NH/π interaction between the DNA base (cytosine and adenine) and the vicinal ring of tpy can be observed as well as the interaction between the CH of tpy and the oxygen of guanine (CH/O interaction) or between the CH_3_ group of timine and the π system of the tpy ring in proximity to such a base (CH/π interaction).

To some extent, also the free energy values calculated from MD runs can be rationalized in terms of the interactions just described for the different structures of **Ru**-DNA adducts. Indeed, after a better electronic description of the systems in QM calculations, the structures that show the major interaction between **Ru** complex and the surrounding nucleotides are those for which the binding free energy obtained from MD simulations are the most favored, **Ru-1** and **Ru-2b**. Similarly, **Ru-III** and **Ru-IVa** exhibit minor interactions and accordingly such arrangements are less stable.

In order to ascertain the photosensitization activity of **Ru** complex also upon intercalation, TDDFT calculations have been performed on each adduct just described. The outcomes are collected in Tables S6 and S7 and Figure S10. As already reported in the literature, the intercalation of the drug between two base pairs should entail a bathochromic shift and hypochromicity of the band in the UV–visible region.^15,21,28,82^ This can be due to the effect of base pairs that could act like electrons donors in the interaction with the intercalated drug and to the decreasing in the energy gap between molecular orbitals (HOMO and LUMO) after DNA binding of the intercalative agent. Analyzing the data reported in Table S6 and Figure S10 it can be noted a hypochromicity of the lowest energy band in all the considered models **Ru-1** to **Ru-IV**. A very slight red-shift of the whole lowest energy band, instead, has been found only for some arrangements and in none of these a participation of the nucleobases in the charge transfer band has been observed. Again, this behavior not in line with that of other intercalative agents can be imputed to the different interactions found for **Ru** complex with respect to other intercalators, whose interaction with DNA causes an enlargement of the intercalation site, instead of just the opening of the pocket observed in the studied case. However, the unaltered absorption spectrum of **Ru** complex suggests the photosensitizing ability should remain equally effective upon intercalation ensuring the PDT action of the drug. Indeed, from triplet states energy collected in Table S7 for all the intercalated arrangements, it can be inferred that the same S_1_ deactivation pathways should occur even upon intercalation.

## 4 Conclusions

A comprehensive computational exploration of the anticancer action of a Ru(II)-based complex is reported here. The structural and electronic features allow the complex to exert a combined anticancer activity as ^1^O_2_ generator for PDT application and intercalator between the DNA nucleobases to induce DNA damage. To assess the mechanism of action of the Ru complex, DFT and TDDFT have been used to simulate the spectroscopic properties of the complex while MD simulations have been exploited to study the interaction with the DNA. The outcomes of quantum mechanism calculations, that are maximum absorption wavelength, triplet states energy and amplitude of the spin–orbit coupling matrix elements, confirm that the complex is able to efficiently generate ^1^O_2_ as several pathways for S_1_ deactivation are energetically accessible and should be kinetically fast, while other types of ROS cannot be produced. The exploration of all the possible intercalation sites evidence the CG/GC site as the arrangement in which the drug establishes a major number of electrostatic interactions. However, even considering all the intercalation arrangements, the spectroscopic properties of the Ru complex remain essentially unaltered upon intercalation; the maximum absorption remains upper to 600 nm and no influence of the nucleobases on the electronic transitions has been found.

These findings evidence the **Ru** complex can exert synergistic anticancer effect, causing DNA damage by reversible intercalation and cell death by ^1^O_2_ photosensitization, and may stimulate the design of multi-action Ru(II)-based complexes.

## Supporting Information Available

Additional details of TDDFT and MD calculations (PDF)
