# Supplementary material for: Computational Assessment of a Dual-Action Ru(II)-Based Complex: Photosensitizer in Photodynamic Therapy and Intercalating Agent for Inducing DNA Damage
Source: Inorg Chem. 2023 May 29;62(23):8948–59. doi: 10.1021/acs.inorgchem.3c00592 (PMC10265706; doi:10.1021/acs.inorgchem.3c00592)
Supplement: Supplementary file 1 — ic3c00592_si_001.pdf [file ic3c00592_si_001.pdf]

Supporting Information

Computational Assessment of a Dual-action Ru(II)-based Complex: Photosensitizer in Photodynamic Therapy and Intercalating Agent for Inducing DNA damage.

Fortuna Ponte,* Stefano Scoditti, Pierraffaele Barretta, Gloria Mazzone*

Department of Chemistry and Chemical Technologies, University of Calabria, 87036 Rende, Italy

*fortuna.ponte@unical.it, gloria.mazzone@unical.it

Table of Contents

- **Figure S1.** Absolute deviation of each exchange and correlation functional from the experimental values of the lowest energy absorption (1.88 eV) S3
- **Discussion on benchmark** S3
- **Table S1**. TD-M06/6-31G(d)/SDD excitation energies ΔE, eV, absorption wavelength λ, nm, oscillator strength *f*, MO contribution %, for the bands in the region 400-700 nm of **Ru** complex........ S4
- **Figure S2.** Decomposition analysis for the first sixteen singlet excited states of Ru complex decomposed into contributions from MC, LC, MLCT, LMCT and LLCT S4
- **Figure S3.** Natural Transition Orbitals (NTOs) for lowest energy transitions as collected in Table S2 of **Ru** complex. S5
- **Figure S4.** a. Decomposition analysis for the 17-150 singlet excited states of **Ru** complex, accounting for bands III and IV, decomposed into contributions from MC, LC, MLCT, LMCT and LLCT. Only the contributions for electronic transitions with oscillator strength greater than 0.01 have been provided. b. Oscillator strength for the 17-150 states S6
- **Table S2**. TD-M06/6-31G(d)/SDD excitation energies ΔE, ev, MO contribution % and theoretical assignment for triplet states of **Ru** complex located below S4 singlet state........ S7
- **Figure S5.** Decomposition analysis for the first six triplet excited states of **Ru** complex decomposed into contributions from MC, LC, MLCT, LMCT and LLCT S7
- **Figure S6.** NTOs for the four triplet states lying below the state located at 2.02 eV........ S8
- **Table S3.** SOC values (cm^{-1}) for the Sn → Tm (with n = 1-4 and m = 1-6) radiationless transitions and singlet– triplet energy gaps (eV) computed for **Ru** complex S9
- **Table S4.** Type I photoreactions. VEA and VIP values (eV) computed in water for $^3O_2$ and **Ru** complex in its ground $[Ru]^+$ and triplet excited $^3[Ru]^+$ states S10
- **Figure S7.** RMSD plots of DNA, four residues around the intercalated agent and Ru complex obtained by MD simulations S11
- **Table S5.** MM-PBSA calculations of **Ru** complex within the four intercalation sites **1-4** and that of ligand L_i within site **1**. Total energies and relative contributions are in kcal mol^{-1} S12
- **Figure S8.** Most representative structure of B-DNA dodecamer with the L_i intercalated in CG/GC active site (**1**) out of clustering analysis performed on the 100 ns long MD S13

- **Figure S9.** RMSD plots of DNA, four residues around the intercalated agent and ligand L_i obtained by MD simulations S14
- **Table S6.** Vertical excitation energies, ΔE (eV), λ_{max} (nm), oscillator strengths, f, and main transitions computed in water for the extracted structures by clustering **Ru-I-IV** S15
- **Figure S10.** Computed absorption spectra in water for all the structural arrangements **Ru-I-IV** derived from the intercalation dynamics of **Ru** complex in the different intercalation sites **1-4** S16
- **Table S7.** Excitation energies ΔE, eV and MO contribution % for triplet states for the intercalated models **Ru-I-IV** located below the bright state S17
- **Ligand L_i and Complex Ru Parameters** S18
- **References** S26

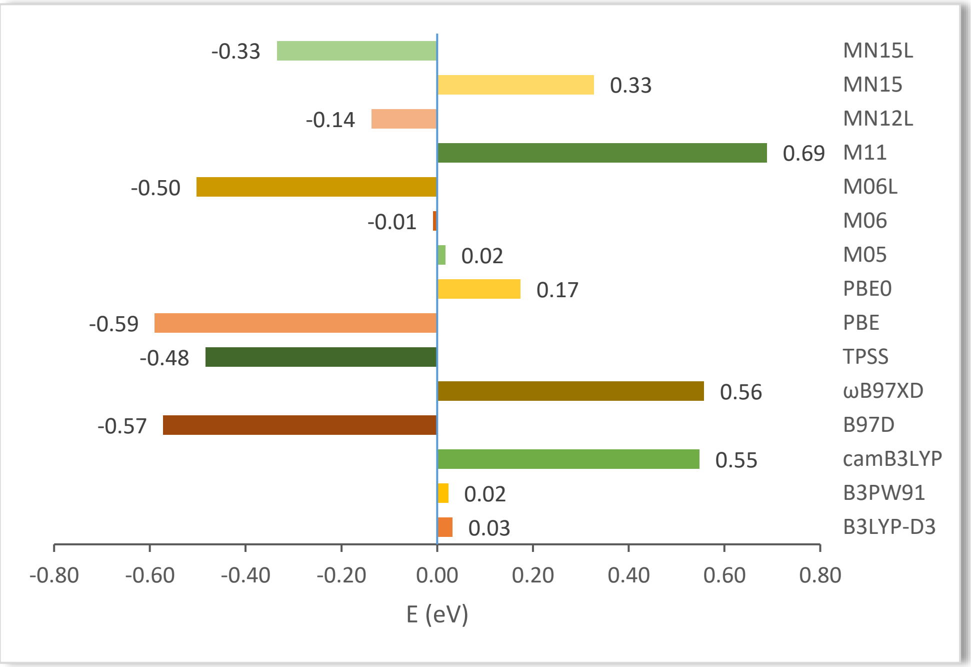

Figure S1. Absolute deviation of each exchange and correlation functional from the experimental value of the lowest energy absorption (1.88 eV)[1].

Discussion on benchmark

The exchange and correlation functionals used for reproducing the maximum absorption wavelength of **Ru** complex are of different type: the GGA (general gradient approximation) functionals B97D[2], and PBE[3,4], the metaNGA (nonseparable gradient approximations) MN15[5], the meta-GGA M06L[6], MN15L[7] and MN12L[8], the global-hybrid GGA B3LYP[9,10], PBE0[11], and B3PW91[12], the global-hybrid meta-GGA M05,[13] M06[14], M11[15] and TPSS[16] and the range-separated hybrid GGA, CAM-B3LYP[17] and ωB97XD[18]. From data reported in Figure S1 it can be inferred that some functionals strongly failed in reproducing the λ_{max}, see for instance B97D. While hybrid functionals like B3LYP and B3PW91 essentially give the same results, overestimating the excitation energy by no more than 0.03 eV. The range separated functionals underestimate it by about 0.6 eV. Even the Minnesota functionals return very diversified results; while the global-hybrid meta-GGA M05 and M06 reproduces the excitation energy with a very little error of 0.01 and 0.02 eV, respectively, a severe overestimation (MN15, M11) or underestimation (MN15L, M06L) has been found employing the others. However, the result of the benchmark study returns the M06 as the most suitable in reproducing one of the main properties for PDT application. Thus, it has been selected for the complete characterization of the absorption spectrum and to perform spin-orbit matrix elements.

Table S1: TD-M06/6-31G(d)/SDD excitation energies ΔE, eV, absorption wavelength λ, nm, oscillator strength *f*, MO contribution %, for the bands in the region 400-700 nm of **Ru** complex.

Band	State	ΔE	λ	*f*	MO contribution	Theoretical Assignment
I	1	1.87	663	0.130	H→L, 61%; H-1→L, 32%	ML$_i$CT
	2	1.93	641	0.001	H-2→L, 49%; H→L+1, 30%	
	3	1.99	622	0.005	H-2→L, 40%; H→L+1, 30%	
	4	2.02	614	0.299	H-1→L, 61%; H→L, 32%	
	5	2.15	578	0.011	H-1→L+1, 52%; H→L+1, 28%	
II	6	2.39	520	0.006	H-2→L+1, 49%	ML$_i$CT/L$_i$C
	7	2.48	500	0.000	H→L+2, 61%	
	8	2.49	497	0.004	H-1→L+3, 33%	
	9	2.58	480	0.017	H-1→L+6, 32%, H-2→L+2, 25%	
	10	2.61	475	0.008	H-2→L+6, 55%, H-1→L+2, 20%	
	11	2.66	466	0.029	H-2→L+2, 51%; H→L+6, 29%	
	12	2.72	456	0.164	H→L+3, 63%	
	13	2.81	442	0.137	H-1→L+2, 40%, H-3→L, 22%	
	14	2.83	439	0.177	H-3→L, 65%	
	15	2.90	428	0.002	H-2→L+3, 88%	
	16	3.05	407	0.237	H-1→L+3, 64%	

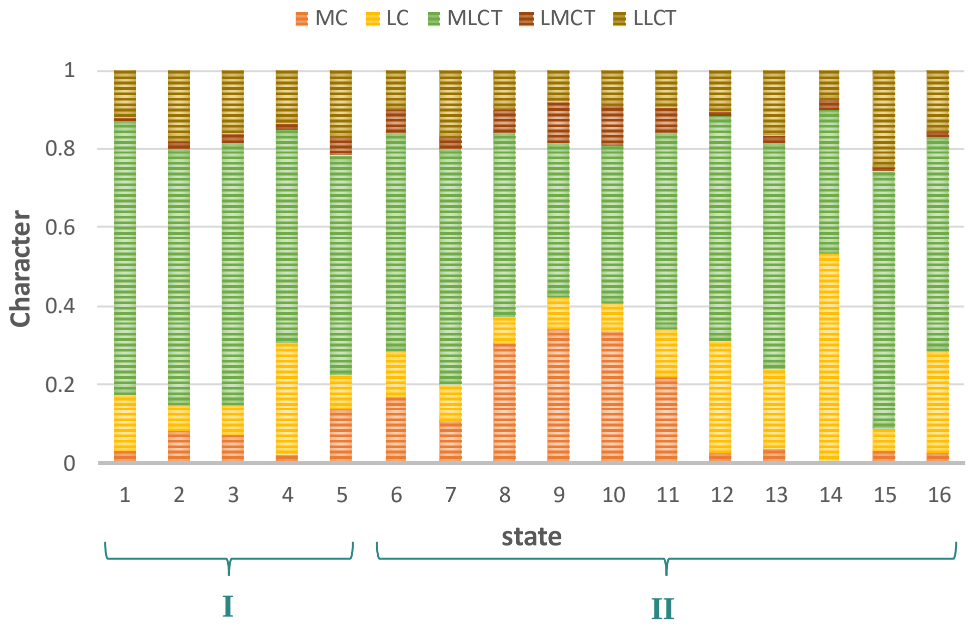

Figure S2. Decomposition analysis made with TheoDORE software[19] for the first sixteen singlet excited states of **Ru** complex decomposed into contributions from MC, LC, MLCT, LMCT and LLCT.

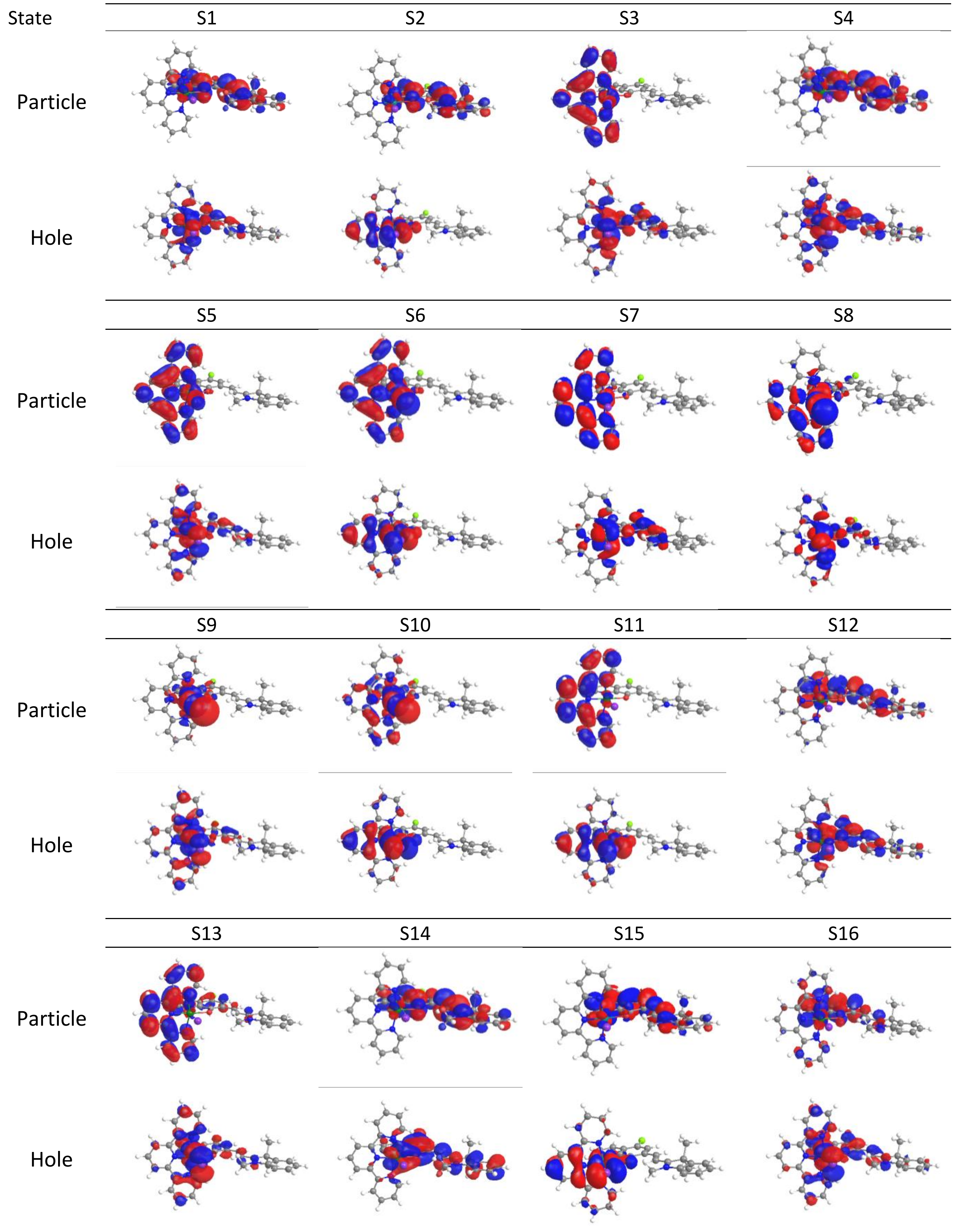

Figure S3. Natural Transition Orbitals for lowest energy transitions as collected in Table S2 of **Ru** complex. Plots have been generated with Chemissian software v4.67.[20]

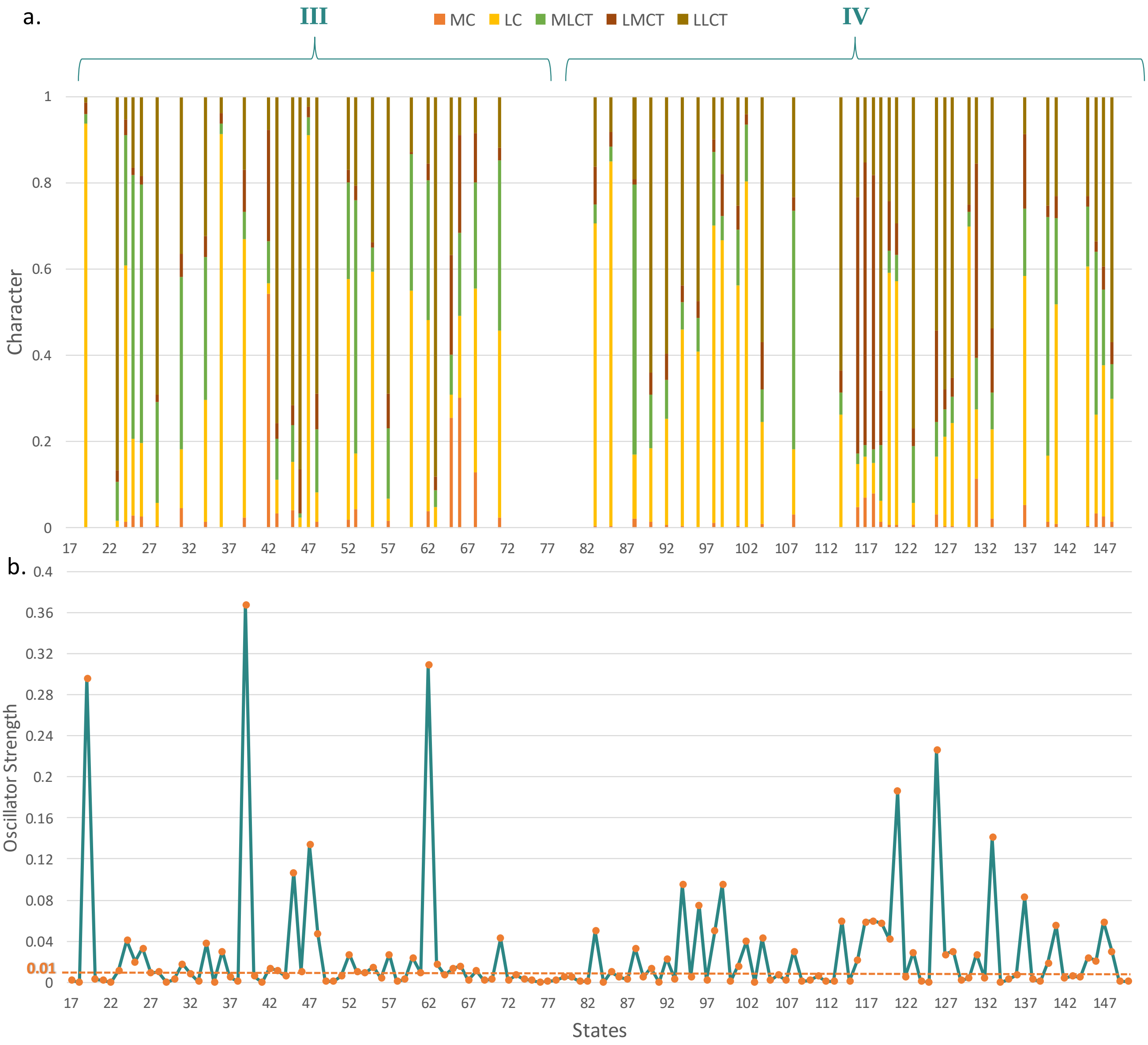

Figure S4. a. Decomposition analysis for the 17-150 singlet excited states of **Ru** complex, accounting for bands III and IV, decomposed into contributions from MC, LC, MLCT, LMCT and LLCT. Only the contributions for electronic transitions with oscillator strength greater than 0.01 have been provided. b. Oscillator strength for the 17-150 states.

Table S2: TD-M06/6-31G(d)/SDD excitation energies ΔE, ev, MO contribution % and theoretical assignment for triplet states of **Ru** complex located below S4 singlet state.

State	ΔE	MO contribution	Theoretical Assignment
T1	1.42	H→L, 90%	**L$_i$C**
T2	1.73	H→L+6, 31%,<br>H→L+1, 28%	**MC/ML$_{tpy}$CT**
T3	1.78	H-2→L+1, 83%	**ML$_{tpy}$CT**
T4	1.85	H-1→L, 78%	**ML$_i$CT**
T5	1.92	H-2→L, 64%<br>H-1→L+1, 23%	**ML$_i$CT**
T6	1.94	H-1→L+1, 43 %<br>H-2→L, 25%	**ML$_{tpy}$CT**

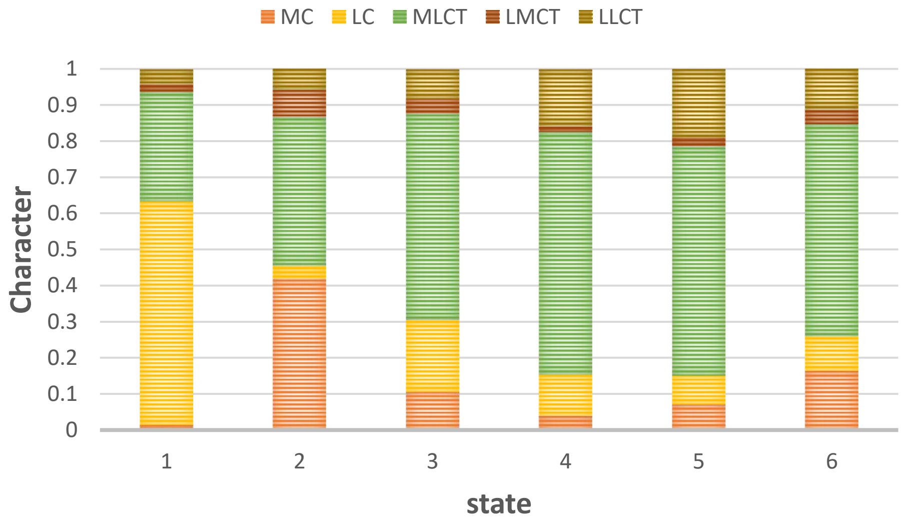

Figure S5. Decomposition analysis for the first six triplet excited states of **Ru** complex decomposed into contributions from MC, LC, MLCT, LMCT and LLCT.

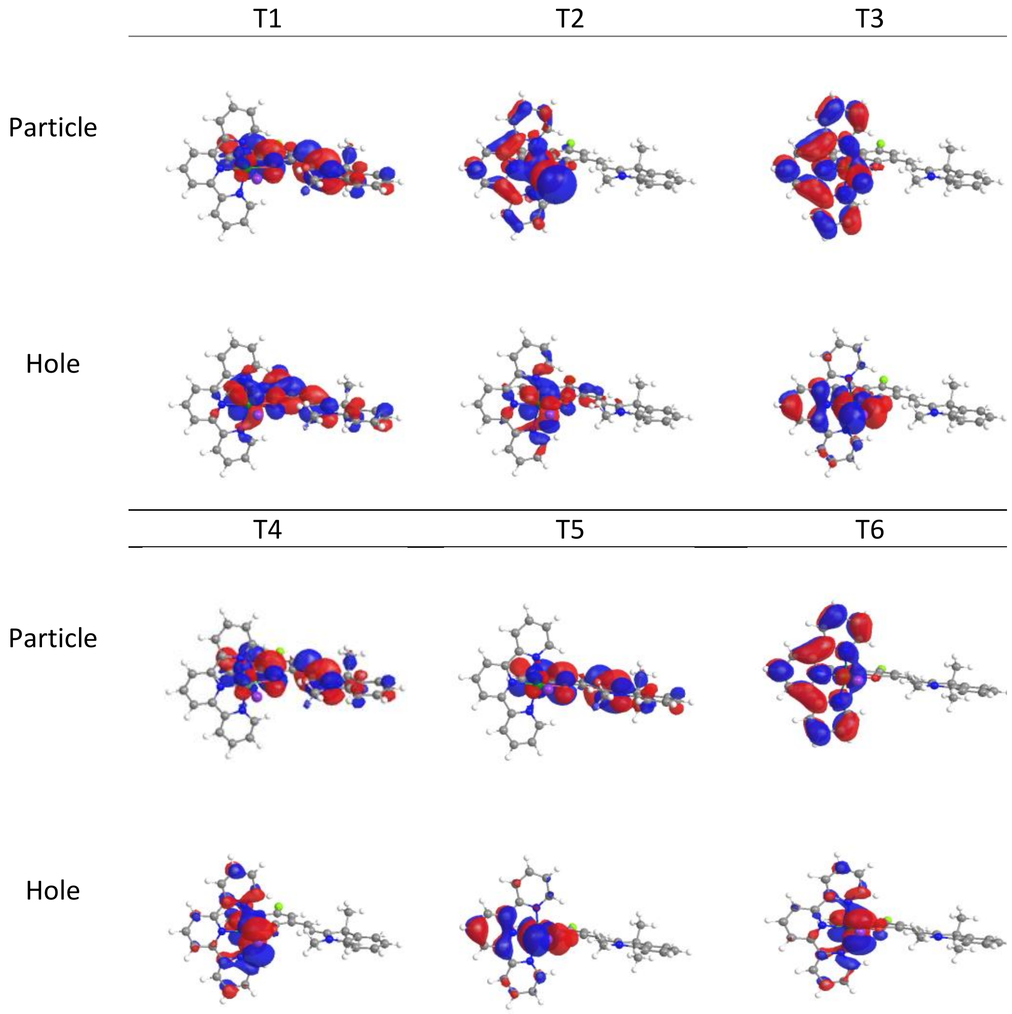

Figure S6. NTOs for the four triplet states lying below the state located at 2.02 eV. Plots have been generated with Chemissian software v4.67.

Table S3. SOC values (cm^{-1}) for the Sn → Tm (with n = 1-4 and m = 1-6) radiationless transitions and singlet– triplet energy gaps (eV) computed for **Ru** complex.

	**S1**		**S2**		**S3**		**S4**	
	SOC	*ΔE*	*SOC*	*ΔE*	*SOC*	*ΔE*	*SOC*	*ΔE*
**T1**	187.4	0.39	199.3	0.46	39.5	0.52	51.16	0.54
**T2**	144.7	0.12	627.1	0.19	248.6	0.24	241.5	0.27
**T3**	67.3	0.01	292	0.08	367.2	0.13	122.0	0.16
**T4**			135.8	0.07	29.37	0.12	423.8	0.15
**T5**			35.54	0.05	75.25	0.10	147.9	0.13
**T6**					323.6	0.06	131.3	0.09

Table S4: Type I photoreactions. VEA and VIP values (eV) computed in water for $^3O_2$[a] and **Ru** complex in its ground $[Ru]^+$ and triplet excited $^3[Ru]^+$ states.

		$[Ru]^+$		$^3[Ru]^+$			
	**Photoprocess**	VEA	VIP	VEA	VIP	**Requirement**	
a.	$^3[Ru]^+ + [Ru]^+ \rightarrow [Ru]^{2+\bullet} + [Ru]^\bullet$	-3.08	4.72	-4.46	3.34	VEA ($^3[Ru]^+$) + VIP ($[Ru]^+$) < 0	✗
b.	$^3[Ru]^+ + {}^3[Ru]^+ \rightarrow [Ru]^{2+\bullet} + [Ru]^\bullet$					VEA ($^3[Ru]^+$) + VIP ($^3[Ru]^+$) < 0	✓
c.	$^3[Ru]^+ + {}^3O_2 \rightarrow [Ru]^{2+\bullet} + O_2^{\cdot(-)}$					VEA ($^3O_2$) + VIP ($^3[Ru]^+$) < 0	✗
d.	$[Ru]^\bullet + {}^3O_2 \rightarrow [Ru]^+ + O_2^{\cdot(-)}$					VEA ($^3O_2$) - VEA ($[Ru]^+$) <0	✗

a. VEA O_2 = -2.30 eV computed at the M06/6-31G* level

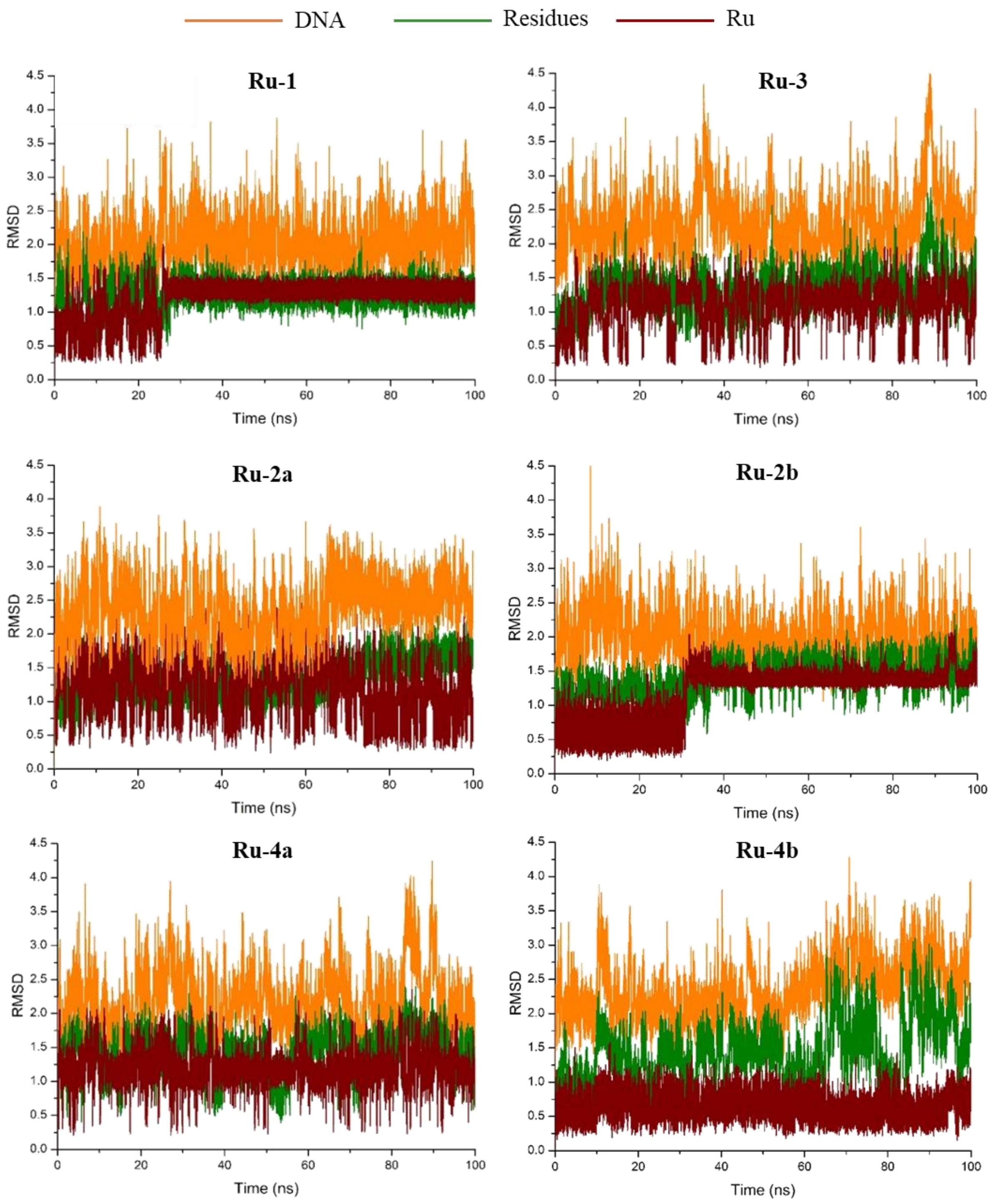

Figure S7. RMSD plots of DNA, four residues around the intercalated agent and Ru complex obtained by MD simulations.

Table S5: MM-PBSA calculations of **Ru** complex within the four intercalation sites **1-4** and that of ligand L_i within site **1**. Total energies and relative contributions are in kcal mol^{-1}.

Energy component	**Ru-1**	**Ru-2a**	**Ru-2b**	**Ru-3**	**Ru-4a**	**Ru-4b**	**L_i**
VDWAALS	-45.4	-40.0	-44.5	-42.3	-40.5	-42.0	-39.9
EEL	-462.0	-476.7	-489.5	-489.7	-468.6	-464.3	-477.0
EPB	474.7	489.5	501.4	504.2	482.3	476.9	488.5
ENPOLAR	-25.5	-22.7	-25.7	-24.3	-23.6	-24.7	-29.7
EDISPER	48.4	44.5	48.8	47.9	46.4	47.3	39.8
ΔG gas	-507.4	-516.7	-534.0	-532.0	-509.0	-506.3	-516.9
ΔG solv	497.5	511.2	524.4	527.9	505.0	499.5	507.6
ΔG binding	-9.9	-5.4	-9.6	-4.2	-4.0	-6.7	-9.4

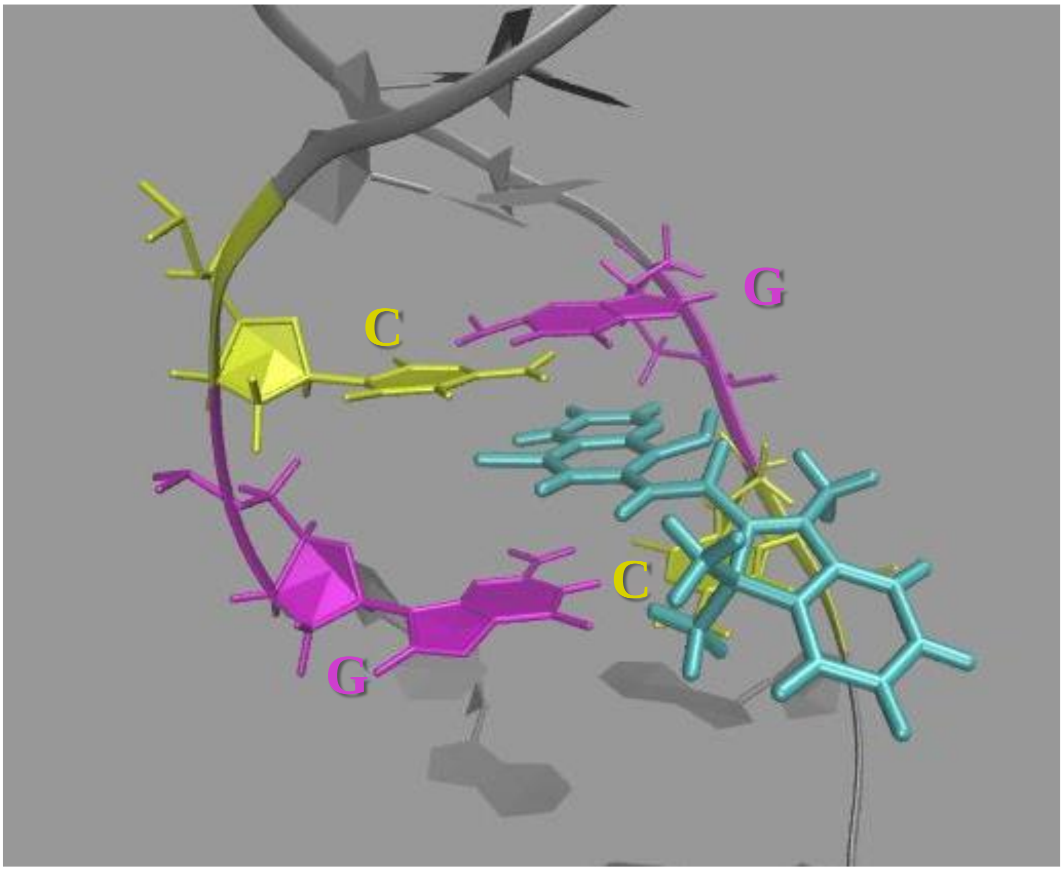

Figure S8. Most representative structure of B-DNA dodecamer with the L_i intercalated in CG/GC active site (**1**) out of clustering analysis performed on the 100 ns long MD.

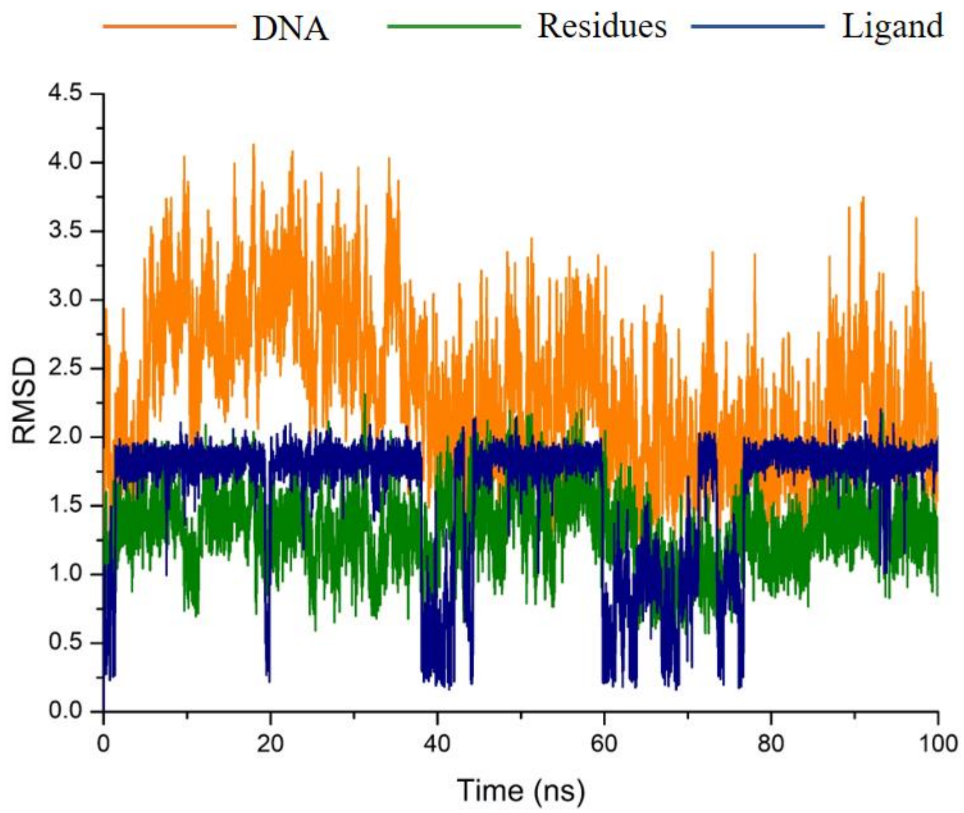

Figure S9. RMSD plots of DNA, four residues around the intercalated agent and ligand L_i obtained by MD simulations.

Table S6: Vertical excitation energies, ΔE (eV), λ_{max} (nm), oscillator strengths, *f*, and main transitions computed in water for the extracted structures by clustering **Ru-I-IV**.

	Band	ΔE	λ	*f*	MO contribution
**Ru-I**	I	1.88	660	0.137	H→L, 66%; H-1→L 26%
		2.00	620	0.093	H-2→L, 32%; H-1→L, 30%
		2.02	615	0.114	H-2→L, 37%; H-1→L, 35%
		2.13	583	0.022	H+1→L+1, 51%; H→L+1, 27%
	II	2.67	464	0.082	H→L+3, 26%; H→L+10, 22%; H-2→L+2, 10%
		2.71	457	0.102	H→L+10, 21%; H→L+3, 16%; H-3→L, 16%
		2.79	444	0.056	H-1→L+2, 28%; H-1→L+3,15%; H-2→L+1, 13%
**Ru-IIa**	I	1.86	666	0.160	H→L, 60%; H-1→L 30%
		1.93	642	0.042	H-2→L, 54%; H-1→L, 22%
		1.96	631	0.141	H-1→L 40%; H→L, 23%
		2.02	615	0.021	H→L+1 42%; H-2→L 18%
	II	2.64	470	0.094	H-2→L+2, 27% ; H→L+3 21%
		2.71	457	0.177	H→L+3 21%; H-4→L 15%; H-1→L+2 13%
		2.74	452	0.078	H-3→54%; H-4→L 21%
		2.77.	448	0.119	H-3→L 43%; H-4→L 32%
**Ru-IIb**	I	1.85	669	0.109	H→L, 60%; H-1→L, 33%
		1.93	643	0.106	H-1→L, 49%; H→L, 24%
		1.97	630	0.056	H→L+1, 36%; H-1→L+1, 16%; H-2→L, 1%
		2.13	582	0.022	H-1→L+1, 31%; H-2→L+1, 30%; H→L+1, 19%
	II	2.61	476	0.038	H-2→L+2, 21%; H-1→L+11, 18%; H-2→L+11, 12%
		2.62	473	0.021	H-1→L+2, 44%; H→L+11, 14%
		2.65	468	0.151	H→L+3, 48%; H-1→L+3, 16%
		2.72	456	0.170	H-4→ L, 70%
		2.77	447	0.077	H-2→L+2, 37%; H-1→L+3, 14%; H-4→L, 12%
**Ru-III**	I	1.92	646	0.227	H→L, 69%; H-1 → L, 12%
		1.97	631	0.073	H→L+1, 46%; H→L, 19%; H-1→L, 17%
		2.02	614	0.029	H-1→L, 62%; H→L+1, 24%
		2.09	593	0.018	H-2→L, 78%
		2.13	581	0.021	H-2→L+1, 60%; H-1→L+1, 10%
	II	2.64	470	0.030	H-1→L+2 55%; H→L+10, 12%
		2.72	456	0.269	H→L+3, 49%; H-3→L, 20%
		2.78	448	0.174	H-3→L, 72%; H→L+3, 17%
**Ru-IVa**	I	1.87	663	0.187	H→L, 68%; H-1→L, 19%
		1.93	642	0.051	H-1→L, 43%; H-2→L, 24%
		1.98	626	0.085	H-2→L, 32%; H-1→L, 29%
		2.01	615	0.022	H→L+1, 36%; H-2→L, 34%
	II	2.64	469	0.094	H-1→L+2, 30%; H-2→L+2, 22%; H→L+10, 14%
		2.70	459	0.243	H-5→L, 37%; H→L+3, 14%; H-4→L, 10%
		2.76	449	0.069	H→L+3, 31%; H-5→L, 31%
**Ru-IVb**	I	1.91	648	0.191	H→L, 75%
		1.99	622	0.103	H-1→L, 42%; H→L, 17%; H→L+1, 17%
		2.13	582	0.025	H-2→L+1, 37%; H-1→L+1, 25%
	II	2.61	475	0.045	H-1→L+2, 29%; H→L+10, 19%
		2.71	458	0.072	H-4→L, 71%
		2.72	456	0.105	H→L+3, 52%; H-5→L, 22%; H-4→L, 16%
		2.76	449	0.109	H-3→L, 31%; H-5→L, 23%; H-2→L+2, 17%
		2.77	448	0.048	H-3→L, 67%; H-5→L, 13%

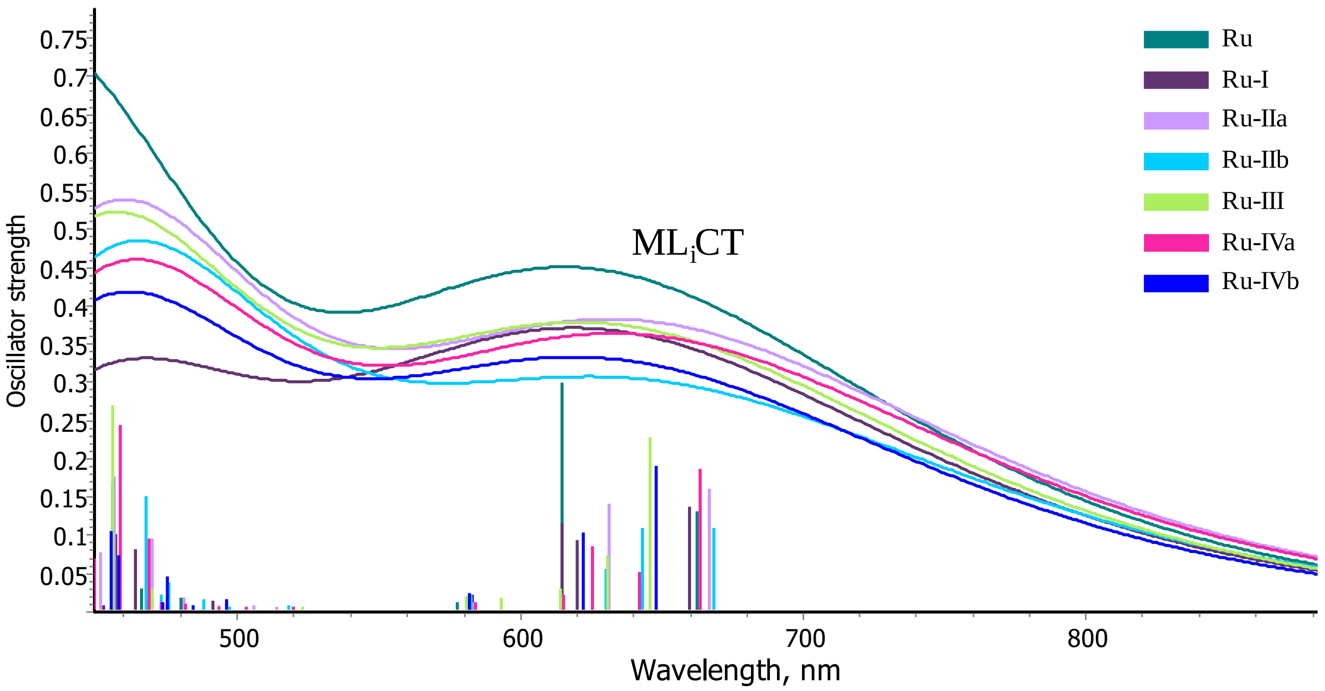

Figure S10. Computed absorption spectra in water for all the structural arrangements **Ru-I-IV** derived from the intercalation dynamics of **Ru** complex in the different intercalation sites **1-4**.

Table S7: Excitation energies ΔE, eV and MO contribution % for triplet states for the intercalated models **Ru-I-IV** located below the bright state.

	State	ΔE	MO contribution	Theoretical Assignment
**Ru-I**	**T1**	1.40	H → L, 90%	ML_iCT
	**T2**	1.76	H-2 → L+1, 26%; H → L+1, 23%; H-1 → L+1, 20%	$ML_{tpy}CT/L_iL_{tpy}CT$
	**T3**	1.79	H-2 → L+1, 54%; H → L+1, 13%	$ML_{tpy}CT/L_iL_{tpy}CT$
	**T4**	1.85	H-1 → L, 77%	ML1CT/LL1CT
**Ru-IIa**	**T1**	1.37	H → L, 90%	ML_iCT
	**T2**	1.75	H-1 → L+1, 29%; H → L+1, 22%; H-2 → L+1, 20%	$ML_{tpy}CT/L_iL_{tpy}CT$
	**T3**	1.81	H-2 → L+1, 31%; H → L+10, 12%; H → L+1, 9%	$ML_{tpy}CT/LL_{tpy}CT$
	**T4**	1.82	H-1 → L, 64%; H-1 → L+3, 8%	ML_iCT/LL_iCT
**Ru-IIb**	**T1**	1.39	H → L, 90%	ML_iCT
	**T2**	1.72	H-1→L+1, 27%; H→L+1 20%; H→L+11 12%	$ML_{tpy}CT/LL_{tpy}CT$
	**T3**	1.78	H-1→L+1, 40%; H-2→L+1 22%	$ML_{tpy}CT/LL_{tpy}CT$
	**T4**	1.80	H-1→L, 79%	ML_iCT/LL_iCT
**Ru-III**	**T1**	1.41	H → L, 89%	ML_iCT
	**T2**	1.71	H → L+1, 27%; H → L+10, 21%; H-1 → L+1, 19%	$ML_{tpy}CT$
	**T3**	1.77	H-1 → L+1, 60%; H → L+10, 11%	$ML_{tpy}CT/L_1L_{tpy}CT$
**Ru-IVa**	**T1**	1.38	H → L, 90%	ML_iCT
	**T2**	1.73	H-1 → L+1, 31%; H → L+1, 27%; H → L+10, 14%	$ML_{tpy}CT/LL_{tpy}CT$
	**T3**	1.78	H-1 → L+1, 31%; H-2 → L+1, 29%	$ML_{tpy}CT/LL_{tpy}CT$
	**T4**	1.85	H-1 → L, 59%; H-2 → L, 21%;	ML_iCT/LL_iCT
**Ru-IVb**	**T1**	1.45	H → L, 88%	ML_iCT
	**T2**	1.74	H-1 → L+1, 44%; H → L+1, 14%	$ML_{tpy}CT/L_iL_{tpy}CT$
	**T3**	1.78	H-1 → L+1, 31%; H → L+1, 20%; H-2 → L+1, 15%	$ML_{tpy}CT/LL_{tpy}CT$
	**T4**	1.89	H-1 → L, 73%	ML_iCT/LL_iCT

Ligand Parameters

a) MOL

```
   0    0    2

This is a remark line
molecule.res
MOL   INT  0
CORRECT     OMIT DU   BEG
  0.0000
   1  DUMM  DU    M    0  -1  -2     0.000      .0        .0      .00000
   2  DUMM  DU    M    1   0  -1     1.449      .0        .0      .00000
   3  DUMM  DU    M    2   1   0     1.523   111.21       .0      .00000
   4  C5    ca    M    3   2   1     1.540   111.208  -180.000 -0.275979
   5  C4    ca    S    4   3   2     1.358   112.744   178.627 -0.035226
   6  H2    ha    E    5   4   3     1.073   120.824     0.540  0.153549
   7  H3    ha    E    4   3   2     1.073     8.385    -4.764  0.185703
   8  C1    ca    M    4   3   2     1.415   127.936    -0.821  0.280308
   9  H1    ha    E    8   4   3     1.075   120.157    -0.537  0.099444
  10  N1    nb    M    8   4   3     1.293   122.736   179.351 -0.533435
  11  C2    ca    M   10   8   4     1.349   118.568     0.104  0.315822
  12  C6    ca    S   11  10   8     1.430   115.380   179.957  0.201773
  13  O1    oh    S   12  11  10     1.324   117.687     0.012 -0.424209
  14  H20   ho    E   13  12  11     0.959   108.015    -0.043  0.374767
  15  C3    ca    M   11  10   8     1.397   123.674    -0.102  0.067872
  16  C7    ca    M   15  11  10     1.429   118.113  -179.928 -0.126574
  17  Cl1   cl    E   16  15  11     1.737   119.947  -179.985 -0.046448
  18  C8    ca    M   16  15  11     1.347   120.341    -0.009 -0.137231
  19  H4    ha    E   18  16  15     1.074   119.046  -179.993  0.163350
  20  C9    ca    M   18  16  15     1.433   122.462    -0.036 -0.075944
  21  C10   ce    M   20  18  16     1.449   117.087   179.945  0.006506
  22  H5    ha    E   21  20  18     1.071   113.055    -0.377  0.195630
  23  C11   ce    M   21  20  18     1.351   128.624   179.682 -0.269493
  24  H6    ha    E   23  21  20     1.067   118.975    -0.378  0.134369
  25  C12   ce    M   23  21  20     1.433   124.014  -179.913  0.237533
  26  N2    nb    S   25  23  21     1.308   120.641  -176.829  0.051868
  27  C20   c3    3   26  25  23     1.464   125.534     2.225 -0.279448
  28  H11   h1    E   27  26  25     1.081   110.261   -76.287  0.148262
  29  H12   h1    E   27  26  25     1.079   110.001    45.326  0.148262
  30  H13   h1    E   27  26  25     1.076   108.676   163.801  0.148262
  31  C13   c3    M   25  23  21     1.535   129.401     4.318  0.171656
  32  C21   c3    3   31  25  23     1.543   111.639    60.226 -0.305232
  33  H14   hc    E   32  31  25     1.081   112.440   -68.920  0.100187
  34  H15   hc    E   32  31  25     1.084   111.278    52.928  0.100187
  35  H16   hc    E   32  31  25     1.083   108.904   171.717  0.100187
  36  C22   c3    3   31  25  23     1.544   111.467   -65.758 -0.305232
  37  H17   hc    E   36  31  25     1.083   108.893  -171.120  0.100187
  38  H18   hc    E   36  31  25     1.084   111.221   -52.380  0.100187
  39  H19   hc    E   36  31  25     1.082   112.573    69.571  0.100187
  40  C15   ca    M   31  25  23     1.515   100.480   177.427  0.032401
  41  C14   ca    M   40  31  25     1.378   109.479     0.649  0.054945
  42  C16   ca    M   41  40  31     1.379   122.919  -179.366 -0.213910
  43  H7    ha    E   42  41  40     1.073   123.002   179.115  0.185569
  44  C18   ca    M   42  41  40     1.389   116.928    -0.415 -0.121578
  45  H9    ha    E   44  42  41     1.074   119.196   179.958  0.167897
  46  C19   ca    M   44  42  41     1.387   121.067     0.071 -0.109664
  47  H10   ha    E   46  44  42     1.074   119.628   179.996  0.161175
  48  C17   ca    M   46  44  42     1.390   120.683     0.187 -0.214634
  49  H8    ha    E   48  46  44     1.075   120.060   179.777  0.186194


LOOP
   C3   C4
   C9   C6
```

```
 C14  N2
 C17  C15

IMPROPER
  C4  C1  C5  H3
  C3  C5  C4  H2
  C5  H1  C1  N1
  C6  C3  C2  N1
  C2  C9  C6  O1
  C2  C7  C3  C4
  C3  C8  C7  Cl1
  C7  C9  C8  H4
  C6  C8  C9  C10
  C9  C11 C10  H5
 C10  C12 C11  H6
 C13  C11 C12  N2
 C20  C14  N2  C12
 C13  C14 C15  C17
 C15  C16 C14  N2
 C14  C18 C16  H7
 C16  C19 C18  H9
 C18  C17 C19  H10
 C15  C19 C17  H8

DONE
STOP
```

b) frcmod file

```
remark goes here
MASS

BOND
ce-nb  582.40   1.287      same as ce-n2
nb-c3  334.70   1.456      same as c3-nc

ANGLE
ha-ca-nb   52.980    116.000   same as ha-ca-n2
ce-ce-nb   69.330    118.930   same as ce-ce-n2
ce-nb-ca   71.110    118.180   same as c2-n2-c2
ce-nb-c3   65.510    118.670   same as c3-n2-ce
c3-ce-nb   66.450    122.730   same as c3-ce-n2
ca-nb-c3   66.260    115.050   same as c3-n2-ca
nb-c3-h1   50.080    108.570   same as h1-c3-nc

DIHE
ca-ca-ce-ha   1   6.650    180.000     2.000     same as X -c2-ce-X
ca-ca-ce-ce   1   6.650    180.000     2.000     same as X -c2-ce-X
ce-ce-c3-ca   1   0.000      0.000     2.000     same as X -c2-c3-X
ce-ce-c3-c3   1   0.000      0.000     2.000     same as X -c2-c3-X
ce-ce-nb-ca   1   4.150    180.000     2.000     same as X -c2-n2-X
ce-ce-nb-c3   1   4.150    180.000     2.000     same as X -c2-n2-X
ce-nb-c3-h1   1   0.000      0.000     3.000     same as X -c3-n2-X
c3-ce-nb-ca   1   4.150    180.000     2.000     same as X -c2-n2-X
c3-ce-nb-c3   1   4.150    180.000     2.000     same as X -c2-n2-X
ca-nb-c3-h1   1   0.000      0.000     3.000     same as X -c3-n2-X
ca-c3-ce-nb   1   0.000      0.000     2.000     same as X -c2-c3-X
nb-ce-c3-c3   1   0.000      0.000     2.000     same as X -c2-c3-X

IMPROPER
ca-ha-ca-nb     1.1     180.0     2.0     General improper torsional angle (2 general atom types)
ca-ca-ca-nb     1.1     180.0     2.0     Using default value
ca-ca-ca-ca     1.1     180.0     2.0     Using default value
ca-ca-ca-ha     1.1     180.0     2.0     General improper torsional angle (2 general atom types)
```

```
ca-ca-ca-oh     1.1     180.0     2.0     Using default value
ca-ca-ca-ce     1.1     180.0     2.0     Using default value
ca-ce-ce-ha     1.1     180.0     2.0     Using default value
ce-ce-ce-ha     1.1     180.0     2.0     Using default value
c3-ce-ce-nb     1.1     180.0     2.0     Using default value
```

Complex Parameters

a) LI0

```
   0    0    2

This is a remark line
molecule.res
LI0   INT  0
CORRECT     OMIT DU   BEG
  0.0000
   1  DUMM  DU    M    0  -1  -2     0.000      .0       .0     .00000
   2  DUMM  DU    M    1   0  -1     1.449      .0       .0     .00000
   3  DUMM  DU    M    2   1   0     1.523   111.21      .0     .00000
   4  I15   Y1    M    3   2   1     1.540   111.208  -180.000 -0.457759


LOOP

IMPROPER

DONE
STOP
```

b) LI1

```
   0    0    2

This is a remark line
molecule.res
LI1   INT  0
CORRECT     OMIT DU   BEG
  0.0000
   1  DUMM  DU    M    0  -1  -2     0.000      .0       .0     .00000
   2  DUMM  DU    M    1   0  -1     1.449      .0       .0     .00000
   3  DUMM  DU    M    2   1   0     1.523   111.21      .0     .00000
   4  N16   Y2    M    3   2   1     1.540   111.208  -180.000  0.132673
   5  C17   ca    M    4   3   2     1.344   117.192   162.217 -0.025641
   6  H18   h4    E    5   4   3     1.085   115.298   175.614  0.123442
   7  C19   ca    M    5   4   3     1.391   122.448    -3.678 -0.164009
   8  H20   ha    E    7   5   4     1.084   119.826   179.979  0.160875
   9  C21   ca    M    7   5   4     1.396   118.770     0.277 -0.069760
  10  H22   ha    E    9   7   5     1.085   120.691   179.688  0.150372
  11  C23   ca    M    9   7   5     1.392   119.072     0.192 -0.133237
  12  H24   ha    E   11   9   7     1.084   120.264   179.312  0.145662
  13  C25   cp    M   11   9   7     1.396   119.631    -0.209  0.022244
  14  C26   cp    M   13  11   9     1.474   124.228   179.040  0.013515
  15  C33   ca    B   14  13  11     1.395   127.209    -0.091 -0.167859
  16  C31   ca    B   15  14  13     1.397   119.193  -178.846 -0.093844
  17  C29   ca    S   16  15  14     1.397   120.109     0.380 -0.165216
  18  H30   ha    E   17  16  15     1.084   120.265   178.985  0.165134
  19  H32   ha    E   16  15  14     1.085   120.002   179.728  0.153595
  20  H34   ha    E   15  14  13     1.084   120.636     0.488  0.165587
  21  N27   Y3    M   14  13  11     1.360   113.151  -179.536  0.266349
  22  C28   cp    M   21  14  13     1.361   122.261   177.934  0.007659
  23  C35   cp    M   22  21  14     1.475   113.163  -177.875  0.022795
```

```
  24  C36   ca    M   23  22  21    1.396   124.235   179.451 -0.131282
  25  H37   ha    E   24  23  22    1.084   120.124     0.606  0.145922
  26  C38   ca    M   24  23  22    1.393   119.625  -178.974 -0.070989
  27  H39   ha    E   26  24  23    1.085   120.243   179.755  0.150597
  28  C40   ca    M   26  24  23    1.397   119.049     0.225 -0.164511
  29  H41   ha    E   28  26  24    1.085   121.374   179.402  0.160742
  30  C42   ca    M   28  26  24    1.391   118.781    -0.143 -0.025218
  31  H43   h4    E   30  28  26    1.085   122.260   178.918  0.123607
  32  N44   Y4    M   30  28  26    1.343   122.420    -0.362  0.133126


LOOP
 C25  N16
 C28  C29
 N44  C35

IMPROPER
 C19  H18  C17  N16
 C17  C21  C19  H20
 C23  C19  C21  H22
 C21  C25  C23  H24
 C23  C26  C25  N16
 C33  C25  C26  N27
 C31  C26  C33  H34
 C33  C29  C31  H32
 C31  C28  C29  H30
 C29  C35  C28  N27
 C36  C28  C35  N44
 C38  C35  C36  H37
 C36  C40  C38  H39
 C38  C42  C40  H41
 C40  H43  C42  N44

DONE
STOP
```

c) LI2

```
   0    0    2

This is a remark line
molecule.res
LI2   INT  0
CORRECT     OMIT DU   BEG
  0.0000
   1  DUMM  DU    M    0  -1  -2    0.000     .0       .0     .00000
   2  DUMM  DU    M    1   0  -1    1.449     .0       .0     .00000
   3  DUMM  DU    M    2   1   0    1.523   111.21      .0     .00000
   4  C69   ca    M    3   2   1    1.540   111.208  -180.000 -0.129409
   5  C71   ca    B    4   3   2    1.399    33.397   -89.181 -0.092831
   6  C68   ca    S    5   4   3    1.398   121.246    26.965 -0.208001
   7  H72   ha    E    6   5   4    1.084   120.437  -179.949  0.162343
   8  H74   ha    E    5   4   3    1.086   119.620  -153.024  0.141117
   9  H73   ha    E    4   3   2    1.086   147.110  -135.687  0.144812
  10  C67   ca    M    4   3   2    1.401    90.123    67.865 -0.209902
  11  H70   ha    E   10   4   3    1.087   120.500   165.573  0.165949
  12  C65   ca    M   10   4   3    1.387   118.660   -14.457  0.116369
  13  C80   c3    B   12  10   4    1.516   130.654   179.997  0.020724
  14  C81   c3    3   13  12  10    1.547   110.438   -61.704 -0.104367
  15  H82   hc    E   14  13  12    1.094   109.267    61.634  0.042741
  16  H83   hc    E   14  13  12    1.094   111.738  -178.636  0.042741
  17  H84   hc    E   14  13  12    1.094   110.984   -57.543  0.042741
  18  C85   c3    3   13  12  10    1.548   110.402    61.826 -0.104308
  19  H86   hc    E   18  13  12    1.093   109.284   -61.753  0.042682
```

```
 20  H87   hc   E   18  13  12    1.094   110.973    57.405  0.042682
 21  H88   hc   E   18  13  12    1.093   111.703   178.499  0.042682
 22  C66   ca   M   12  10   4    1.398   120.020     0.015  0.065972
 23  N75   nb   M   22  12  10    1.417   108.461   179.999 -0.013134
 24  C76   c3   3   23  22  12    1.466   124.299  -179.943 -0.081492
 25  H77   h1   E   24  23  22    1.093   110.572   119.719  0.092543
 26  H78   h1   E   24  23  22    1.094   110.574  -120.573  0.092543
 27  H79   h1   E   24  23  22    1.088   109.478    -0.460  0.092543
 28  C64   ce   M   23  22  12    1.348   111.613    -0.041  0.124930
 29  C61   ce   M   28  23  22    1.404   120.769  -179.972 -0.033486
 30  H63   ha   E   29  28  23    1.081   117.349    -0.031 -0.028009
 31  C60   ce   M   29  28  23    1.387   125.974   179.982 -0.213189
 32  H62   ha   E   31  29  28    1.087   118.842    -0.043  0.216119
 33  C46   ca   M   31  29  28    1.420   126.287   179.920 -0.023911
 34  C49   ca   M   33  31  29    1.433   119.337   179.976 -0.072581
 35  H51   ha   E   34  33  31    1.086   118.300    -0.017  0.130634
 36  C58   ca   M   34  33  31    1.367   122.975   179.964 -0.127796
 37  Cl1   cl   E   36  34  33    1.755   119.596  -179.974 -0.053633
 38  C89   ca   M   36  34  33    1.437   121.136    -0.066  0.090506
 39  C47   ca   M   38  36  34    1.413   125.538  -179.921 -0.099818
 40  H50   ha   E   39  38  36    1.084   119.832    -0.054  0.149972
 41  C57   ca   M   39  38  36    1.383   119.337  -179.982 -0.128823
 42  H48   ha   E   41  39  38    1.085   120.801  -179.996  0.162168
 43  C56   ca   M   41  39  38    1.403   119.995    -0.078 -0.041862
 44  H45   ha   E   43  41  39    1.084   121.629  -179.948  0.104978
 45  N54   Y6   M   43  41  39    1.336   122.012     0.069 -0.138335
 46  C55   ca   M   45  43  41    1.371   118.846     0.004  0.111250
 47  C52   ca   M   46  45  43    1.446   115.016   179.993  0.097364
 48  O53   Y5   M   47  46  45    1.283   117.736    -0.086 -0.052177


LOOP
 C66  C68
 C64  C80
 C52  C46
 C55  C89

IMPROPER
 C71  C67  C69  H73
 C69  C68  C71  H74
 C66  C71  C68  H72
 C65  C69  C67  H70
 C80  C66  C65  C67
 C65  C68  C66  N75
 C64  C76  N75  C66
 C80  C61  C64  N75
 C64  C60  C61  H63
 C46  C61  C60  H62
 C52  C49  C46  C60
 C46  C58  C49  H51
 C89  C49  C58  Cl1
 C47  C55  C89  C58
 C89  C57  C47  H50
 C47  C56  C57  H48
 C57  H45  C56  N54
 C52  C89  C55  N54
 C55  C46  C52  O53

DONE
STOP
```

d) Ru

```
  0   0   2
```

```
This is a remark line
molecule.res
RU    INT  0
CORRECT     OMIT DU   BEG
  0.0000
   1  DUMM  DU    M    0  -1  -2    0.000      .0       .0     .00000
   2  DUMM  DU    M    1   0  -1    1.449      .0       .0     .00000
   3  DUMM  DU    M    2   1   0    1.523   111.21      .0     .00000
   4  RU    M1    M    3   2   1    1.540   111.208  -180.000 -0.156613


LOOP

IMPROPER

DONE
STOP
```

e) frcmod file

```
REMARK GOES HERE, THIS FILE IS GENERATED BY MCPB.PY
MASS
M1 101.07                  Ru ion
Y1 126.9       4.690       Iodine
Y2 14.01       0.530       Sp2 N in pure aromatic systems
Y3 14.01       0.530       Sp2 N in pure aromatic systems
Y4 14.01       0.530       Sp2 N in pure aromatic systems
Y5 16.00       0.465       Ether and ester oxygen
Y6 14.01       0.530       Sp2 N in pure aromatic systems

BOND
M1-Y1   50.8    2.7634     Created by Seminario method using MCPB.py
M1-Y2   59.5    2.0947     Created by Seminario method using MCPB.py
M1-Y3  128.2    1.9574     Created by Seminario method using MCPB.py
M1-Y4   59.6    2.0947     Created by Seminario method using MCPB.py
M1-Y5   57.7    2.1290     Created by Seminario method using MCPB.py
M1-Y6   64.8    2.1066     Created by Seminario method using MCPB.py
Y2-ca  488.0    1.3390       SOURCE3_SOURCE5    6806              0.0055
Y2-cp  488.5    1.3387       SOURCE4_SOURCE5     190              0.0068
Y6-ca  488.0    1.3390       SOURCE3_SOURCE5    6806              0.0055
ca-Y4  488.0    1.3390       SOURCE3_SOURCE5    6806              0.0055
ca-Y5  376.6    1.3696       SOURCE1_SOURCE5    6900              0.0064
cp-Y3  488.5    1.3387       SOURCE4_SOURCE5     190              0.0068
cp-Y4  488.5    1.3387       SOURCE4_SOURCE5     190              0.0068
ce-nb  582.40   1.287      same as ce-n2
nb-c3  334.70   1.456      same as c3-nc

ANGL
M1-Y2-ca   191.42    128.02    Created by Seminario method using MCPB.py
M1-Y2-cp   178.38    112.97    Created by Seminario method using MCPB.py
M1-Y3-cp   133.75    119.02    Created by Seminario method using MCPB.py
M1-Y4-ca   191.22    128.02    Created by Seminario method using MCPB.py
M1-Y4-cp   178.21    112.97    Created by Seminario method using MCPB.py
M1-Y5-ca   104.42    115.76    Created by Seminario method using MCPB.py
M1-Y6-ca   140.84    120.65    Created by Seminario method using MCPB.py
Y2-M1-Y1    90.43     89.62    Created by Seminario method using MCPB.py
Y3-M1-Y1    91.63     91.97    Created by Seminario method using MCPB.py
Y3-M1-Y2   134.56     79.50    Created by Seminario method using MCPB.py
Y4-M1-Y1    90.40     89.62    Created by Seminario method using MCPB.py
Y4-M1-Y2   160.40    158.94    Created by Seminario method using MCPB.py
Y4-M1-Y3   134.54     79.50    Created by Seminario method using MCPB.py
Y5-M1-Y1    71.44     90.97    Created by Seminario method using MCPB.py
Y5-M1-Y2   116.32    100.54    Created by Seminario method using MCPB.py
```

Y5-M1-Y3 109.39 177.05 Created by Seminario method using MCPB.py
Y5-M1-Y4 116.25 100.51 Created by Seminario method using MCPB.py
Y6-M1-Y1 85.18 167.74 Created by Seminario method using MCPB.py
Y6-M1-Y2 125.42 92.60 Created by Seminario method using MCPB.py
Y6-M1-Y3 144.77 100.28 Created by Seminario method using MCPB.py
Y6-M1-Y4 125.42 92.59 Created by Seminario method using MCPB.py
Y6-M1-Y5 100.07 76.77 Created by Seminario method using MCPB.py
Y2-ca-ca 68.83 122.94 SOURCE3_SOURCE5 5507 1.1495
Y2-ca-h4 51.88 116.03 SOURCE3_SOURCE5 2217 0.2861
Y2-cp-ca 69.01 121.62 SOURCE4_SOURCE5 174 0.6998
Y2-cp-cp 68.20 116.61 SOURCE4_SOURCE5 235 1.1595
Y4-ca-h4 51.88 116.03 SOURCE3_SOURCE5 2217 0.2861
Y4-cp-ca 69.01 121.62 SOURCE4_SOURCE5 174 0.6998
ca-Y6-ca 68.35 117.22 SOURCE3_SOURCE5 3343 1.0306
ca-ca-Y4 68.83 122.94 SOURCE3_SOURCE5 5507 1.1495
ca-ca-Y5 69.58 119.20 SOURCE3 52 0.5240
ca-ca-Y6 68.83 122.94 SOURCE3_SOURCE5 5507 1.1495
ca-cp-Y3 69.01 121.62 SOURCE4_SOURCE5 174 0.6998
cp-Y2-ca 68.11 118.05 SOURCE4_SOURCE5 160 0.7542
cp-Y3-cp 68.110 118.050 same as ca-nb-cp
cp-Y4-ca 68.11 118.05 SOURCE4_SOURCE5 160 0.7542
cp-cp-Y3 68.20 116.61 SOURCE4_SOURCE5 235 1.1595
cp-cp-Y4 68.20 116.61 SOURCE4_SOURCE5 235 1.1595
ha-ca-Y6 52.980 116.000 same as ha-ca-n2
ha-ca-nb 52.980 116.000 same as ha-ca-n2
ce-ce-nb 69.330 118.930 same as ce-ce-n2
ce-nb-ca 71.110 118.180 same as c2-n2-c2
ce-nb-c3 65.510 118.670 same as c3-n2-ce
c3-ce-nb 66.450 122.730 same as c3-ce-n2
ca-nb-c3 66.260 115.050 same as c3-n2-ca
nb-c3-h1 50.080 108.570 same as h1-c3-nc

DIHE
X -Y2-ca-X 2 9.6 180.0 2.0 same as X-CA-NC-X
X -Y4-ca-X 2 9.6 180.0 2.0 same as X-CA-NC-X
X -ca-Y6-X 2 9.6 180.0 2.0 same as X-CA-NC-X
M1-Y2-ca-ca 3 0.00 0.00 3.0 Treat as zero by MCPB.py
M1-Y2-ca-h4 3 0.00 0.00 3.0 Treat as zero by MCPB.py
M1-Y2-cp-ca 3 0.00 0.00 3.0 Treat as zero by MCPB.py
M1-Y2-cp-cp 3 0.00 0.00 3.0 Treat as zero by MCPB.py
M1-Y3-cp-ca 3 0.00 0.00 3.0 Treat as zero by MCPB.py
M1-Y3-cp-cp 3 0.00 0.00 3.0 Treat as zero by MCPB.py
M1-Y4-ca-ca 3 0.00 0.00 3.0 Treat as zero by MCPB.py
M1-Y4-ca-h4 3 0.00 0.00 3.0 Treat as zero by MCPB.py
M1-Y4-cp-ca 3 0.00 0.00 3.0 Treat as zero by MCPB.py
M1-Y4-cp-cp 3 0.00 0.00 3.0 Treat as zero by MCPB.py
M1-Y5-ca-ca 3 0.00 0.00 3.0 Treat as zero by MCPB.py
M1-Y6-ca-ca 3 0.00 0.00 3.0 Treat as zero by MCPB.py
M1-Y6-ca-ha 3 0.00 0.00 3.0 Treat as zero by MCPB.py
Y3-M1-Y2-ca 3 0.00 0.00 3.0 Treat as zero by MCPB.py
Y3-M1-Y2-cp 3 0.00 0.00 3.0 Treat as zero by MCPB.py
Y4-M1-Y2-ca 3 0.00 0.00 3.0 Treat as zero by MCPB.py
Y4-M1-Y2-cp 3 0.00 0.00 3.0 Treat as zero by MCPB.py
Y4-M1-Y3-cp 3 0.00 0.00 3.0 Treat as zero by MCPB.py
Y5-M1-Y2-ca 3 0.00 0.00 3.0 Treat as zero by MCPB.py
Y5-M1-Y2-cp 3 0.00 0.00 3.0 Treat as zero by MCPB.py
Y5-M1-Y3-cp 3 0.00 0.00 3.0 Treat as zero by MCPB.py
Y5-M1-Y4-ca 3 0.00 0.00 3.0 Treat as zero by MCPB.py
Y5-M1-Y4-cp 3 0.00 0.00 3.0 Treat as zero by MCPB.py
Y6-M1-Y2-ca 3 0.00 0.00 3.0 Treat as zero by MCPB.py
Y6-M1-Y2-cp 3 0.00 0.00 3.0 Treat as zero by MCPB.py
Y6-M1-Y3-cp 3 0.00 0.00 3.0 Treat as zero by MCPB.py
Y6-M1-Y4-ca 3 0.00 0.00 3.0 Treat as zero by MCPB.py
Y6-M1-Y4-cp 3 0.00 0.00 3.0 Treat as zero by MCPB.py
Y6-M1-Y5-ca 3 0.00 0.00 3.0 Treat as zero by MCPB.py

ca-Y2-M1-Y1 3 0.00 0.00 3.0 Treat as zero by MCPB.py
ca-Y4-M1-Y1 3 0.00 0.00 3.0 Treat as zero by MCPB.py
ca-Y4-M1-Y2 3 0.00 0.00 3.0 Treat as zero by MCPB.py
ca-Y4-M1-Y3 3 0.00 0.00 3.0 Treat as zero by MCPB.py
ca-Y4-cp-ca 1 4.8 180.0 2.0 same as X -ca-nb-X
ca-Y5-M1-Y1 3 0.00 0.00 3.0 Treat as zero by MCPB.py
ca-Y5-M1-Y2 3 0.00 0.00 3.0 Treat as zero by MCPB.py
ca-Y5-M1-Y3 3 0.00 0.00 3.0 Treat as zero by MCPB.py
ca-Y5-M1-Y4 3 0.00 0.00 3.0 Treat as zero by MCPB.py
ca-Y6-M1-Y1 3 0.00 0.00 3.0 Treat as zero by MCPB.py
ca-Y6-M1-Y2 3 0.00 0.00 3.0 Treat as zero by MCPB.py
ca-Y6-M1-Y3 3 0.00 0.00 3.0 Treat as zero by MCPB.py
ca-Y6-M1-Y4 3 0.00 0.00 3.0 Treat as zero by MCPB.py
ca-Y6-M1-Y5 3 0.00 0.00 3.0 Treat as zero by MCPB.py
ca-cp-Y2-ca 1 4.8 180.0 2.0 same as X -ca-nb-X
ca-cp-Y3-cp 1 4.8 180.0 2.0 same as ca-nb-cp-ca
cp-Y2-M1-Y1 3 0.00 0.00 3.0 Treat as zero by MCPB.py
cp-Y3-M1-Y1 3 0.00 0.00 3.0 Treat as zero by MCPB.py
cp-Y3-M1-Y2 3 0.00 0.00 3.0 Treat as zero by MCPB.py
cp-Y4-M1-Y1 3 0.00 0.00 3.0 Treat as zero by MCPB.py
cp-Y4-M1-Y2 3 0.00 0.00 3.0 Treat as zero by MCPB.py
cp-Y4-M1-Y3 3 0.00 0.00 3.0 Treat as zero by MCPB.py
cp-cp-Y2-ca 1 4.8 180.0 2.0 same as X -ca-nb-X
cp-cp-Y3-cp 1 4.8 180.0 2.0 same as X -ca-nb-X
cp-cp-Y4-ca 1 4.8 180.0 2.0 same as X -ca-nb-X
ca-ca-ce-ha 1 6.650 180.000 2.000 same as X -c2-ce-X
ca-ca-ce-ce 1 6.650 180.000 2.000 same as X -c2-ce-X
ce-ce-c3-ca 1 0.000 0.000 2.000 same as X -c2-c3-X
ce-ce-c3-c3 1 0.000 0.000 2.000 same as X -c2-c3-X
ce-ce-nb-ca 1 4.150 180.000 2.000 same as X -c2-n2-X
ce-ce-nb-c3 1 4.150 180.000 2.000 same as X -c2-n2-X
ce-nb-c3-h1 1 0.000 0.000 3.000 same as X -c3-n2-X
c3-ce-nb-ca 1 4.150 180.000 2.000 same as X -c2-n2-X
c3-ce-nb-c3 1 4.150 180.000 2.000 same as X -c2-n2-X
ca-nb-c3-h1 1 0.000 0.000 3.000 same as X -c3-n2-X
ca-c3-ce-nb 1 0.000 0.000 2.000 same as X -c2-c3-X
nb-ce-c3-c3 1 0.000 0.000 2.000 same as X -c2-c3-X

IMPR
Y2-ca-cp-cp 1.1 180.0 2.0 Using default value
Y6-ca-ca-ca 1.1 180.0 2.0 Using default value
Y4-ca-cp-cp 1.1 180.0 2.0 Using default value
Y6-ca-ca-ha 1.1 180.0 2.0 General improper torsional angle (2 general atom types)
Y4-ca-ca-h4 1.1 180.0 2.0 Using default value
Y2-ca-ca-h4 1.1 180.0 2.0 Using default value
Y3-ca-cp-cp 1.1 180.0 2.0 Using default value
ca-ha-ca-nb 1.1 180.0 2.0 General improper torsional angle (2 general atom types)
ca-ca-ca-nb 1.1 180.0 2.0 Using default value
ca-ca-ca-ca 1.1 180.0 2.0 Using default value
ca-ca-ca-ha 1.1 180.0 2.0 General improper torsional angle (2 general atom types)
ca-ca-ca-oh 1.1 180.0 2.0 Using default value
ca-ca-ca-ce 1.1 180.0 2.0 Using default value
ca-ce-ce-ha 1.1 180.0 2.0 Using default value
ce-ce-ce-ha 1.1 180.0 2.0 Using default value
c3-ce-ce-nb 1.1 180.0 2.0 Using default value

NONB
M1 1.4815 0.0560000000 Adopted from atom type Ru6+2 from UFF (Rappe et al. JACS, 114, 10024)
Y1 2.15 0.50 Junmei, 2010
Y2 1.8240 0.1700 OPLS
Y3 1.8240 0.1700 OPLS
Y4 1.8240 0.1700 OPLS
Y5 1.6837 0.1700 OPLS ether
Y6 1.8240 0.1700 OPLS

References

(1) He, G.; Xu, N.; Ge, H.; Lu, Y.; Wang, R.; Wang, H.; Du, J.; Fan, J.; Sun, W.; Peng, X. Red-Light-Responsive Ru Complex Photosensitizer for Lysosome Localization Photodynamic Therapy. *ACS Appl. Mater. Interfaces* **2021**, *13* (17), 19572–19580. https://doi.org/10.1021/ACSAMI.0C22551.

(2) Grimme, S. Semiempirical GGA-Type Density Functional Constructed with a Long-Range Dispersion Correction. *J Comput Chem* **2006**, *27*, 1787–1799. https://doi.org/10.1002/jcc.20495.

(3) Perdew, J. P.; Burke, K.; Ernzerhof, M. Generalized Gradient Approximation Made Simple. *Phys. Rev. Lett.* **1996**, *77* (18), 3865–3868. https://doi.org/10.1103/PhysRevLett.77.3865.

(4) Perdew, J. P.; Burke, K.; Ernzerhof, M. Generalized Gradient Approximation Made Simple [Phys. Rev. Lett. 77, 3865 (1996)]. *Phys. Rev. Lett.* **1997**, *78* (7), 1396. https://doi.org/10.1103/PhysRevLett.78.1396.

(5) Yu, H. S.; He, X.; Li, S. L.; Truhlar, D. G. MN15: A Kohn–Sham Global-Hybrid Exchange–Correlation Density Functional with Broad Accuracy for Multi-Reference and Single-Reference Systems and Noncovalent Interactions. *Chem. Sci.* **2016**, *7* (8), 5032. https://doi.org/10.1039/C6SC00705H.

(6) Zhao, Y.; Truhlar, D. G. A New Local Density Functional for Main-Group Thermochemistry, Transition Metal Bonding, Thermochemical Kinetics, and Noncovalent Interactions. *J. Chem. Phys.* **2006**, *125* (19), 194101. https://doi.org/10.1063/1.2370993.

(7) Yu, H. S.; He, X.; Truhlar, D. G. MN15-L: A New Local Exchange-Correlation Functional for Kohn–Sham Density Functional Theory with Broad Accuracy for Atoms, Molecules, and Solids. *J. Chem. Theory Comput.* **2016**, *12* (3), 1280–1293. https://doi.org/10.1021/acs.jctc.5b01082.

(8) Peverati, R.; Truhlar, D. G. An Improved and Broadly Accurate Local Approximation to the Exchange–Correlation Density Functional: The MN12-L Functional for Electronic Structure Calculations in Chemistry and Physics. *Phys. Chem. Chem. Phys.* **2012**, *14* (38), 13171. https://doi.org/10.1039/c2cp42025b.

(9) Becke, A. D. Density-functional Thermochemistry. III. The Role of Exact Exchange. *J. Chem. Phys.* **1998**, *98* (7), 5648. https://doi.org/10.1063/1.464913.

(10) Lee, C.; Yang, W.; Parr, R. G. Development of the Colle-Salvetti Correlation-Energy Formula into a Functional of the Electron Density. *Phys. Rev. B* **1988**, *37* (2), 785–789. https://doi.org/10.1103/PhysRevB.37.785.

(11) Adamo, C.; Barone, V. Toward Reliable Density Functional Methods without Adjustable Parameters: The PBE0 Model. *J. Chem. Phys.* **1999**, *110* (13), 6158. https://doi.org/10.1063/1.478522.

(12) Perdew, J. P.; Burke, K. Generalized Gradient Approximation for the Exchange-Correlation Hole of a Many-Electron System. *Phys. Rev. B - Condens. Matter Mater. Phys.* **1996**, *54* (23), 16533–16539. https://doi.org/10.1103/PhysRevB.54.16533.

(13) Zhao, Y.; Schultz, N. E.; Truhlar, D. G. Exchange-Correlation Functional with Broad Accuracy for Metallic and Nonmetallic Compounds, Kinetics, and Noncovalent Interactions. *J. Chem. Phys.* **2005**, *123* (16), 161103. https://doi.org/10.1063/1.2126975.

(14) Zhao, Y.; Truhlar, D. G. The M06 Suite of Density Functionals for Main Group Thermochemistry,

Thermochemical Kinetics, Noncovalent Interactions, Excited States, and Transition Elements: Two New Functionals and Systematic Testing of Four M06-Class Functionals and 12 Other Func. *Theor. Chem. Acc.* **2008**, *120* (1), 215–241. https://doi.org/10.1007/s00214-007-0310-x.

(15) Peverati, R.; Truhlar, D. G. Improving the Accuracy of Hybrid Meta-GGA Density Functionals by Range Separation. *J. Phys. Chem. Lett.* **2011**, *2* (21), 2810–2817. https://doi.org/10.1021/jz201170d.

(16) Tao, J.; Perdew, J. P.; Staroverov, V. N.; Scuseria, G. E. Climbing the Density Functional Ladder: Nonempirical Meta–Generalized Gradient Approximation Designed for Molecules and Solids. *Phys. Rev. Lett.* **2003**, *91* (14), 146401. https://doi.org/10.1103/PHYSREVLETT.91.146401/FIGURES/1/MEDIUM.

(17) Yanai, T.; Tew, D. P.; Handy, N. C. A New Hybrid Exchange–Correlation Functional Using the Coulomb-Attenuating Method (CAM-B3LYP). *Chem. Phys. Lett.* **2004**, *393* (1–3), 51–57. https://doi.org/10.1016/J.CPLETT.2004.06.011.

(18) Chai, J. Da; Head-Gordon, M. Long-Range Corrected Hybrid Density Functionals with Damped Atom-Atom Dispersion Corrections. *Phys. Chem. Chem. Phys.* **2008**, *10* (44), 6615–6620. https://doi.org/10.1039/b810189b.

(19) Plasser, F. TheoDORE: A Toolbox for a Detailed and Automated Analysis of Electronic Excited State Computations. *J. Chem. Phys.* **2020**, *152* (8), 084108. https://doi.org/10.1063/1.5143076.

(20) Skripnikov, L. Chemissian. 2020.
